# Mitigating proton trapping in cubic perovskite oxides via ScO_6_ octahedral networks

**DOI:** 10.1038/s41563-025-02311-w

**Published:** 2025-08-08

**Authors:** Kota Tsujikawa, Junji Hyodo, Susumu Fujii, Kazuki Takahashi, Yuto Tomita, Nai Shi, Yasukazu Murakami, Shusuke Kasamatsu, Yoshihiro Yamazaki

**Affiliations:** 1 Platform of Inter-/Transdisciplinary Energy Research (Q-PIT), Kyushu University, Fukuoka, Japan; 2 INAMORI Frontier Research Center, Kyushu University, Fukuoka, Japan; 3 Department of Materials, Kyushu University, Fukuoka, Japan; 4 Center for Energy Systems Design (CESD), International Institute for Carbon-Neutral Energy Research (WPI-I2CNER), Kyushu University, Fukuoka, Japan; 5 Division of Materials and Manufacturing Science, Osaka University, Suita, Japan; 6 Nanostructures Research Laboratory, Japan Fine Ceramics Center, Nagoya, Japan; 7 Department of Science, Yamagata University, Yamagata, Japan; 8 The Ultramicroscopy Research Center, Kyushu University, Nishi-ku, Fukuoka, Japan; 9 Academic Assembly (Faculty of Science), Yamagata University, Yamagata, Japan

**Keywords:** Fuel cells, Solid-state chemistry

## Abstract

Advances in electrochemical devices have been primarily driven by the discovery and development of electrolyte materials. Yet the development of high-performance and chemically stable proton-conducting oxide electrolytes remains a challenge due to proton trapping and the resulting trade-offs between ionic carrier concentration and conductivity in doped oxides. Here we demonstrate that cubic perovskite oxides with heavy Sc doping can overcome these limitations. BaSn_0.3_Sc_0.7_O_3–*δ*_ and BaTi_0.2_Sc_0.8_O_3–*δ*_ are found to exceed the technological threshold of a total proton conductivity of 0.01 S cm^−1^ for fuel cell electrolytes at 300 °C. The structural stability of BaSn_0.3_Sc_0.7_O_3–*δ*_ is further validated under harsh chemical and fuel cell conditions. Molecular dynamics simulations using a machine learning force field illustrate rapid proton diffusion pathways along the ScO_6_ octahedral network, effectively mitigating proton trapping, while protons are preferentially associated with Sc. Lattice softness is proposed as a primary design descriptor for increasing Sc content in perovskite oxides and developing high-performance electrolytes for electrochemical devices.

## Abstract (web summary)

Solid-state electrolytes that operate at a temperature of 300 °C suffer from a H^+^ concentration–conductivity trade-off. Here heavy Sc doping of cubic perovskites is shown to bypass this trade-off, enabling a proton conductivity of 0.01 S cm^−1^ that arises from rapid proton diffusion along ScO_6_ octahedra.

## Main

The discovery and development of novel electrolyte materials has historically led to advancements in electrochemical devices^1–5^. Examples include lithium-ion conduction in the Li–Ge–P–S system^4^ and proton conduction in SrCe_0.95_Yb_0.05_O_3−*δ*_ perovskite oxide^1^, which have paved the way for all-solid-state batteries^5^, intermediate-temperature proton ceramic fuel cells^6^ and electrolysers for fuel production^7^. An ideal electrolyte should exhibit both high ionic conductivity and enduring chemical and electrochemical stability during device operation. However, meeting these criteria remains a challenge. Liquid electrolytes^8^ offer high ionic conductivity but can compromise device integrity owing to potential leakage. By contrast, a solid electrolyte eliminates such risks but requires overcoming substantial activation barriers for ionic carrier migration. Hydride-conducting compounds^9^ offer a conductivity of 0.01 S cm^−1^ between room temperature and 400 °C but are unstable under oxidizing conditions. Proton-conducting CsH_2_PO_4_ fulfils the requirements within only a narrow operating temperature and water solubility window of 233–254 °C at a water partial pressure of 0.4 atm (ref. ^10^), limiting its broader application. Currently, no stable solid-state and/or protonic electrolytes exist that can operate under fuel cell and electrolyser conditions at 300 °C.

Proton-conducting perovskite oxides^11,12^ exhibit a high proton conductivity of 0.01 S cm^−1^ at 450 °C with strong chemical stability, as reported for Y-doped barium zirconate^2,13^. Perovskite oxides with a cubic or pseudo-cubic structure represented by the general chemical formula ABO_3_, where A and B are cations, are characterized by a network of corner-sharing BO_6_ octahedra with A-cation sublattices. Upon doping with cations of lower valency (that is, acceptor doping), usually on the B site, oxygen vacancies are generated to maintain charge neutrality, as represented using the Kröger–Vink notation:

1 $${{\mathrm{M}}}_{2}{{\rm{O}}}_{3}+2{{\mathrm{B}}}_{{\mathrm{B}}}^{\times }+{\mathrm{O}}_{{\rm{O}}}^{\times }={{\mathrm{v}}}_{{\rm{O}}}^{\bullet \bullet }+2{{\mathrm{M}}}_{{\mathrm{B}}}^{{\prime} }+2{\mathrm{B}}{\mathrm{O}}_{2}.$$

These oxygen vacancies are filled with hydroxyl groups when exposed to moisture (hydration reaction):

2 $${{\mathrm{v}}}_{{\mathrm{O}}}^{{\rm{\bullet }}{\rm{\bullet }}}+{{\mathrm{O}}}_{{\mathrm{O}}}^{\times }+{{\mathrm{H}}}_{2}{\mathrm{O}}\leftrightarrow 2{\mathrm{O}}{{\mathrm{H}}}_{{\mathrm{O}}}^{{\rm{\bullet }}}.$$

The incorporated protons are often strongly associated with acceptor dopants^14^ that reside on the nearest or second nearest neighbour oxygen to the acceptor dopant^14–17^:

3 $${{\mathrm{M}}}_{\mathrm{B}}^{{\prime} }+{\rm{O}}{\mathrm{H}}_{{\rm{O}}}^{{\rm{\bullet }}}={\left({{\mathrm{M}}}_{\mathrm{B}}^{{\prime} }\mathrm{O}{\mathrm{H}}_{{\rm{O}}}^{{\rm{\bullet }}}\right)}^{\times }.$$

Further details of this general defect chemistry for acceptor-doped perovskite oxides can be found in Supplementary Section 1. This association effect acts to stabilize the hydrated state^16^, where the maximum proton content equals the amount of acceptor dopant in the case of a trivalent cation substituting at a tetravalent cation site. The protons can diffuse throughout the material via a combination of rotation and hopping to neighbouring proton sites (Grotthuss mechanism^11^) on the three-dimensional network of BO_6_ octahedra.

**Fig. 1:**
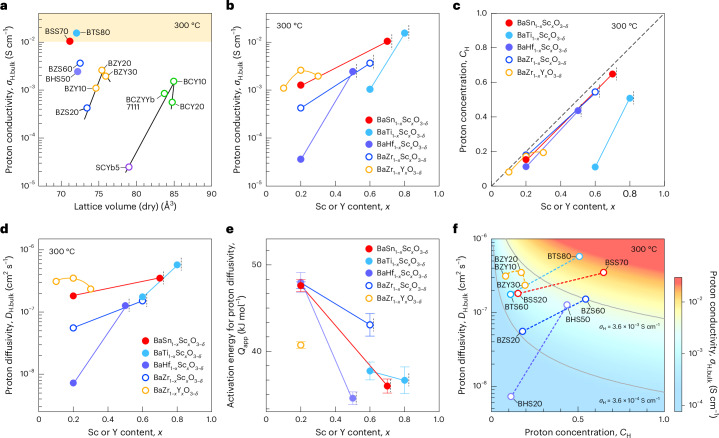
Proton transport properties of Sc-doped barium stannates and barium titanates at 300 °C. **a**, Proton conductivity in bulk (*σ*_H.bulk_) plotted against lattice volume. **b**, Proton conductivity in bulk versus dopant content (*x*). **c**, Proton concentration (*C*_H_) versus *x*. **d**, Proton diffusivity in bulk (*D*_H.bulk_) versus *x*. **e**, Apparent activation energy for proton diffusivity (*Q*_app_). **f**, Proton diffusivity in bulk plotted against proton concentration. The lattice volumes of BSS70 and BTS80 in **a** are derived from Supplementary Table 1. The apparent activation energies in **e** were determined in the temperature range between 200 °C and 300 °C. The yellow region in **a** shows materials that meet the technological threshold^26^ of electrolyte for solid-state electrochemical devices. The vertical dashed lines in **b**–**e** show the solubility limits for Sc in each system. The diagonal dashed line in **c** shows the theoretical maximum proton content. The proton diffusivity for Y-doped barium zirconates in **d** and **f** was obtained using the Nernst–Einstein relationship and reported values for proton conductivity^2,18^, proton concentration^39^ and lattice volume^39^. Error bars in **e** represent the standard error of the slope obtained from the least-squares fits of proton diffusivities in the Arrhenius plots, using 9, 8, 6, 8, 6, 8, 11, 8 and 10 data points for BaSn_0.3_Sc_0.7_O_3__−__*δ*_, BaSn_0.8_Sc_0.2_O_3__−__*δ*_, BaTi_0.2_Sc_0.8_O_3__−__*δ*_, BaTi_0.4_Sc_0.6_O_3−__*δ*_, BaHf_0.5_Sc_0.5_O_3__−__*δ*_, BaHf_0.8_Sc_0.2_O_3__−__*δ*_, BaZr_0.4_Sc_0.6_O_3__−__*δ*_, BaZr_0.8_Sc_0.2_O_3__−__*δ*_ and BaZr_0.8_Y_0.2_O_3__−__*δ*_, respectively. Diffusivity datasets for BaZr_0.4_Sc_0.6_O_3__−__*δ*_ (ref. ^3^), BaZr_0.8_Sc_0.2_O_3__−__*δ*_ (ref. ^3^) and BaZr_0.8_Y_0.2_O_3__−__*δ*_ (ref. ^14^) were reproduced from the respective publications. Bulk proton conductivities (*σ*_H_) in **f** are presented with a corresponding colour bar; the solid contour lines show proton conductivities of 3.6 × 10^−3^ S cm^−1^ and 3.6 × 10^−4^ S cm^−1^. Abbreviations are as follows: BSS70 for BaSn_0.3_Sc_0.7_O_3−*δ*_, BSS20 for BaSn_0.8_Sc_0.2_O_3−*δ*_, BTS80 for BaTi_0.2_Sc_0.8_O_3−*δ*_, BTS60 for BaTi_0.4_Sc_0.6_O_3−*δ*_, BZS60 for BaZr_0.4_Sc_0.6_O_3−*δ*_ (ref. ^3^), BZS20 for BaZr_0.8_Sc_0.2_O_3−*δ*_ (ref. ^3^), BZY20 for BaZr_0.8_Y_0.2_O_3−*δ*_ (refs. ^2,39^), BZY10 for BaZr_0.9_Y_0.1_O_3−*δ*_ (refs. ^18,39^), BZY30 for BaZr_0.7_Y_0.3_O_3−*δ*_ (refs. ^18,39^), SCYb5 for SrCe_0.95_Yb_0.05_O_3−*δ*_ (refs. ^40,41^), BCY10 for BaCe_0.9_Y_0.1_O_3−*δ*_ (refs. ^42,43^), BCY20 for BaCe_0.8_Y_0.2_O_3−*δ*_ (refs. ^42,43^) and BCZYYb7111 for BaCe_0.7_Zr_0.1_Y_0.1_Yb_0.1_O_3−*δ*_ (refs. ^44,45^). The proton contents given for the compounds are determined using thermogravimetry. Source data

The development of proton-conducting perovskite oxides has followed a design principle aimed at enhancing proton conductivity by increasing lattice volume through the incorporation of larger host cations and acceptor dopants within the perovskite structure^11^ (Fig. 1a). However, the proton conductivity in barium zirconate peaks at a Y-doping level of 20 at.% (refs. ^2,18^), substantially below its solubility limit of approximately 50 at.% (ref. ^19^), and any further increase in Y doping reduces its conductivity^20^, which implies that carrier diffusivity also decreases (Fig. 1b,d). This has conventionally been explained as the detrimental effect of the proton–dopant association leading to proton trapping^14,16^. More recent theoretical works have pointed out the formation of deeper proton traps surrounded by Y clusters as the underlying mechanism for the decrease in conductivity at high dopant concentrations^21^. This trade-off is inherent in proton-conducting oxides, where the proton–dopant association stabilizes protons in oxides, enabling the hydration of oxides but impeding proton diffusion in the lattice (proton trapping), as found for proton-conducting barium zirconates^2,14,18,20,22^. A similar phenomenon is also observed for oxide-ion conduction in doped zirconia and ceria^23^.

The recent discovery of superior proton conduction in heavily (60 at.%) Sc-doped barium zirconate^3^ offers a promising avenue for overcoming the trade-off in doped ionic conductors. In BaZr_0.4_Sc_0.6_O_3–*δ*_, the proton concentration, diffusivity and conductivity all increase continuously up to approximately the solubility limit (Fig. 1b–d). It is proposed that heavy acceptor doping facilitates proton diffusion in barium zirconates and analogous materials, based on computations^24^ and experiments^3,25^. These results suggest that heavy Sc doping promises to overcome the trade-off in doped ionic conductors.

In this study we performed systematic searches for Ba-based host perovskite oxides capable of dissolving substantial amounts of Sc. We found that heavy Sc doping, up to 70, 80 and 50 at.%, is possible in barium stannate, barium titanate and barium hafnate, respectively. The resultant total proton conductivities in the barium stannate and barium titanate exceed the technological threshold^26^ of 0.01 S cm^−1^ for fuel cell electrolytes at 300 °C (Fig. 1a), overcoming the well-known and problematic H^+^ concentration–conductivity trade-off in doped oxides. Doped barium stannates^27,28^ and barium titanates^29^ have long been overlooked due to their lower proton conductivities and less favourable hydration behaviour at dopant contents up to ~20 at.%, and proton conductivity investigations under heavy doping close to the solubility limit had not been attempted. Molecular dynamics simulations employing state-of-the-art machine learning force fields reveal that the heavy Sc doping creates proton diffusion pathways along the ScO_6_ octahedral network that mitigate the influence of proton trapping on diffusion. A correlation between reduced bulk modulus and increased ScO_6_ content in the perovskite framework provides a design guideline for developing such electrolytes.

## Heavy Sc doping of BaSnO_3_, BaTiO_3_ and BaHfO_3_ perovskites

The 20 at.% and 70 at.% Sc-doped barium stannates, 60 at.% and 80 at.% Sc-doped barium titanates and 20 at.% and 50 at.% Sc-doped barium hafnates were synthesized using solid-state reactions. Powder X-ray diffraction analysis and Rietveld refinement for dehydrated BaSn_0.3_Sc_0.7_O_3−*δ*_, BaTi_0.2_Sc_0.8_O_3−*δ*_, BaSn_0.8_Sc_0.2_O_3−*δ*_, BaTi_0.4_Sc_0.6_O_3−*δ*_, BaHf_0.5_Sc_0.5_O_3−*δ*_ and BaHf_0.8_Sc_0.2_O_3−*δ*_ (Supplementary Fig. 1) show that these compounds primarily exhibit cubic perovskite phases in the space group ${Pm}\bar{3}m$ (structural parameters are provided in Supplementary Table 1). BaSn_0.3_Sc_0.7_O_3−*δ*_, BaTi_0.2_Sc_0.8_O_3−*δ*_ and BaHf_0.5_Sc_0.5_O_3−*δ*_ contain 5.1 wt%, 2.3 wt% and 5.3 wt%, respectively, of secondary BaSc_2_O_4_. By using inductively coupled plasma optical emission spectrometry (ICP-OES) and considering the secondary phase, the chemical composition of BaSn_0.3_Sc_0.7_O_3−*δ*_ perovskite was determined to be Ba_1.00_Sn_0.311_Sc_0.695_O_3−*δ*_ (Supplementary Table 2). The substitutional acceptor Sc doping and vacancy formation for barium stannates, titanates and hafnates, described in equation (1), are validated by the monotonic increase or decrease of lattice volume upon Sc doping (Supplementary Fig. 2).

## Proton conductivities from a.c. impedance

The a.c. impedance spectroscopy, conducted under a humidified Ar atmosphere, reveals that heavy Sc doping at levels of 70 at.% and 80 at.% in stannate and titanate, respectively, results in fast proton conduction at 300 °C. For 20 at.% Sc-doped barium stannate and 60 at.% Sc-doped barium titanate, the proton conductivity is only 0.001 S cm^−1^ at 300 °C (Fig. 1b and Supplementary Figs. 3–8). However, increasing the Sc content to 70 at.% and 80 at.% enhances the proton conductivity by a factor of ten, reaching 0.01 and 0.016 S cm^−1^, respectively (Fig. 1b). This tenfold increase represents a substantial improvement compared to the Y-doped barium zirconate system, which peaks at a dopant level of 20 at.% Y (refs. ^2,18^). Within the Sc-doped systems, barium stannate outperforms barium hafnate and barium zirconate^3^ in proton conductivity for all Sc contents by a factor of 2–30 and 2–4, respectively (Fig. 1b). The temperature dependence of the proton conductivity in the stannates, titanates and hafnates (Supplementary Fig. 9) also highlights the advantage of heavy Sc doping regardless of operating temperature.

**Fig. 2:**
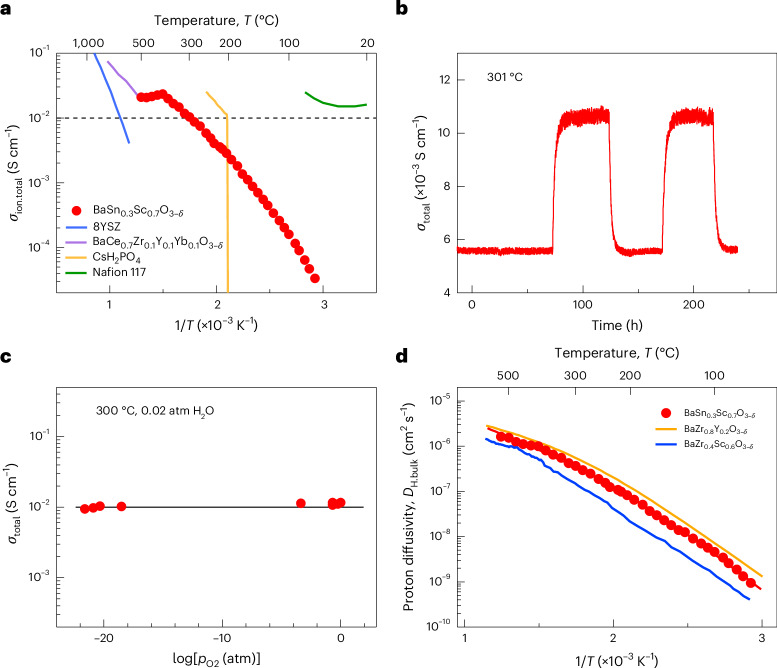
Proton conduction and diffusion in BaSn_0.3_Sc_0.7_O_3–*δ*_. **a**, Comparison of total ionic conductivity (*σ*_ion.total_) with those of conventional electrolytes^10,46,47^ used in fuel cells. *T*, temperature. **b**, H–D isotope effect at 300 °C, shown by total conductivity (*σ*_total_) differences. **c**, Total conductivity plotted against the log of oxygen partial pressure, $p_{{\rm{o}}_2}$, at 300 °C. **d**, Proton diffusivity. The dashed line in **a** shows the technological threshold^26^ of electrolytes for solid-state electrochemical devices. The partial pressure of light and heavy water in **b** was 0.02 atm. For the ab initio molecular dynamics calculations in **d**, deuterium was used instead of hydrogen. The data for 8YSZ (yttria-stabilized zirconia with a mole fraction of 8 mol% Y_2_O_3_ (ref. ^48^)), BaCe_0.7_Zr_0.1_Y_0.1_Yb_0.1_O_3−*δ*_ (ref. ^47^), CsH_2_PO_4_ (ref. ^10^), Nafion 117 (ref. ^46^), BaZr_0.4_Sc_0.6_O_3−*δ*_ (ref. ^3^) and BaZr_0.8_Y_0.2_O_3−*δ*_ (ref. ^14^) were taken from the literature. Source data

BaSn_0.3_Sc_0.7_O_3–*δ*_ and BaTi_0.2_Sc_0.8_O_3–*δ*_ exhibit high total proton conductivities, exceeding 0.01 S cm^−1^, at and above 300 °C under a water partial pressure of 0.02 atm (Fig. 2a and Supplementary Fig. 15). The total conductivity comprises both bulk and grain boundary resistivities in the polycrystalline pellet. Large grains with a mean grain size of 16.7 μm for BaSn_0.3_Sc_0.7_O_3–*δ*_ (Supplementary Fig. 16) minimize the resistive contribution across grain boundaries to the overall proton resistivity in the polycrystalline pellet.

## Proton concentrations from thermogravimetry

The thermogravimetry analysis (Supplementary Fig. 10) provides compelling evidence that the defect chemistry and hydration processes of these materials adhere to the principles governing acceptor-doped perovskite oxide proton conductors, as described by equations (1) and (2). Proton concentration at 300 °C nearly equals the Sc content (Fig. 1c) for all perovskite oxides examined excluding barium titanates, indicating almost complete hydration in those systems. The hydration enthalpies for BaSn_0.3_Sc_0.7_O_3−*δ*_, BaSn_0.8_Sc_0.2_O_3−*δ*_, BaTi_0.2_Sc_0.8_O_3−*δ*_, BaTi_0.4_Sc_0.6_O_3−*δ*_, BaHf_0.5_Sc_0.5_O_3−*δ*_ and BaHf_0.8_Sc_0.2_O_3−*δ*_ are determined by van’t Hoff plots as −125 ± 1, −131 ± 3, −99 ± 5, −71 ± 2, −112 ± 2 and −80 ± 2 kJ mol^−1^, respectively (Supplementary Fig. 11 and Supplementary Table 3), which are in the range of reported values for acceptor-doped perovskite oxides^30,12^. When comparing among heavily Sc-doped samples, barium titanates exhibit a much lower proton concentration (Fig. 1c) and less negative hydration enthalpy (Supplementary Fig. 12) than barium stannate and barium zirconate. In situ Fourier transform infrared (FT-IR) spectroscopy for Sc-doped barium stannates and barium titanates (Supplementary Fig. 13) also provides direct evidence of the hydration reaction of equation (2).

## Proton diffusivities

Sc-doped barium stannate displays high proton diffusivity across a wide range of Sc concentrations. By using the Nernst–Einstein relationship and measuring proton conductivities and concentrations (Fig. 1b,c), the proton diffusivities in barium stannates with 20 at.% and 70 at.% Sc at 300 °C were determined to be 1.9 × 10^−7^ and 3.5 × 10^−7^ cm^2^ s^−1^, respectively (Fig. 1d). These proton diffusivities are 2.3 to 3.4 times greater than those in Sc-doped zirconates^3^, depending on Sc content. By contrast, barium titanates display a steep Sc dependence in proton diffusivity, similar to that observed in barium zirconates. Sc-doped barium hafnate shows the steepest dependence of diffusivity on Sc content, but the Sc solubility of 50 at.% limits the proton diffusivity to 1 × 10^−7^ cm^2^ s^−1^. The temperature-dependent proton diffusivity (Supplementary Fig. 14) in the range between 200 °C and 300 °C provides apparent activation energies, plotted against Sc content in Fig. 1e. In these perovskites, Sc doping lowers the activation energy. The reduced activation energy (Fig. 1e) and enhanced proton diffusivity (Fig. 1d) appear to be a general advantage of heavy Sc doping in the perovskites. As a result, heavy Sc doping overcomes the trade-off between proton diffusivity and proton concentration reported in conventional systems, leading to a high conductivity of 0.01 S cm^−1^ at 300 °C in barium stannate and barium titanate (Fig. 1f).

## Confirmation of proton conduction in BaSn_0.3_Sc_0.7_O_3–*δ*_

An isotope exchange experiment between D_2_O and H_2_O moisture at 301 °C, while maintaining a water partial pressure of 0.02 atm, yielded an isotope effect of 1.95 (Fig. 2b). This strongly suggests that protons are the primary ionic carriers. The higher value (~2) than the square root of the mass ratio between a deuterium and a proton, 1.41, is likely attributable to a more profound influence of the zero-point energy between them^31^ at 301 °C. Recent calculations that incorporate both nuclear quantum effects and anharmonicity report an isotope effect of 1.9 at 227 °C in barium zirconate^32^, which closely aligns with our experimental result in 70 at.% Sc-doped barium stannate at 300 °C. A continuous isotope exchange experiment over 261 h at 301 °C confirmed the chemical stability of proton insertion and extraction in BaSn_0.3_Sc_0.7_O_3–*δ*_ (Fig. 2b).

A constant total conductivity across a wide range of oxygen partial pressures ranging from highly reducing to oxidizing conditions between 10^−22^ and 1 atm at 300 °C (Fig. 2c) indicates a proton transference number close to unity. As proof of ionic conduction, the open circuit voltage for a BaSn_0.3_Sc_0.7_O_3–*δ*_ electrolyte-supported protonic ceramic fuel cell at 300 °C matches the theoretical value (1.194 V) calculated from the Nernst equation for the given conditions (Supplementary Fig. 17). An anode-supported fuel cell, consisting of a thin BaSn_0.3_Sc_0.7_O_3_ electrolyte that includes 5 at.% Ni (Supplementary Fig. 18), shows an open circuit voltage of approximately 1 V at 300 °C (Supplementary Fig. 19 and Supplementary Table 4). The equivalence of the X-ray photoelectron spectra for reduced BaSn_0.3_Sc_0.7_O_3−*δ*_ and undoped BaSnO_3_ (ref. ^33^; Supplementary Fig. 20) offers further evidence that the Sn cation remains tetravalent. All the results, including the isotope effect of Fig. 2b, confirm that BaSn_0.3_Sc_0.7_O_3–*δ*_ is purely proton conductive at 300 °C, fulfilling another crucial requirement for fuel cell electrolytes.

Across a broad temperature spectrum, BaSn_0.3_Sc_0.7_O_3–*δ*_ exhibits a proton diffusivity that is twice as high as that found for state-of-the-art 60 at.% Sc-doped barium zirconate^3^ (Fig. 2d) and is equivalent to that for 20 at.% Y-doped barium zirconate^14^. The nonlinear Arrhenius plot of proton diffusivity for BaSn_0.3_Sc_0.7_O_3–*δ*_ suggests the existence of some sort of proton trapping.

## Proton transport examined by machine learning force field

**Fig. 3:**
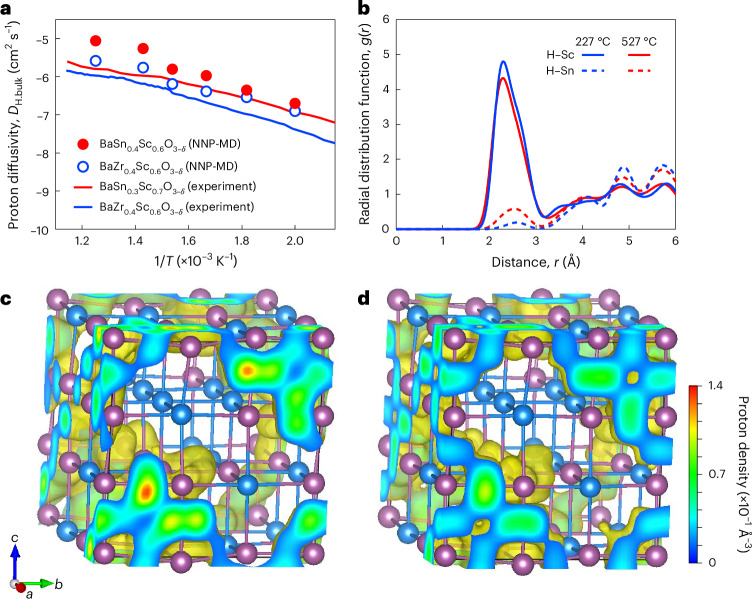
Proton diffusion and its trajectory in 60 at.% Sc-doped barium stannate. **a**, Proton diffusivity from molecular dynamics using neural network potential (NNP-MD) compared to experiment. **b**, Radial distribution function (*g*(*r*), where *r* is distance). **c**,**d**, Proton trajectory at 227 °C (**c**) and 527 °C (**d**). Purple and blue spheres in **c** and **d** denote Sc and Sn cations, respectively. The proton density isosurface, rendered in yellow in **c** and **d**, corresponds to the isosurface level of 1.4 × 10^−2^ Å^−3^. The two-dimensional density maps at the *a*–*b*, *b*–*c* and *c*–*a* cross-sections are also given with the associated colour bar. The experimental^3^ and calculated^12^ data for BaZr_0.4_Sc_0.6_O_3−δ_ in **a** were taken from the literature. Source data

Molecular dynamics simulations using a machine learning force field were performed with realistic dopant configurations determined by Monte Carlo simulations^17^. Diffusivities predicted by the simulations for 60 at.% Sc-doped barium stannate and barium zirconate between 227 °C and 527 °C are in good agreement with the experimental results, although the absolute values are slightly overestimated (Fig. 3a; the data for the zirconate are taken from the literature^12^). The observation of the trajectory of protons in the molecular dynamics simulations confirms the Grotthuss mechanism, a combination of proton rotation around the donor oxygen and hopping between the oxygens (an animation is provided as Supplementary Data 3). A clear tendency of protons to reside near dopant Sc compared with Sn, that is, a proton–dopant association, is evident from the analysis of H–Sc and H–Sn pair distribution functions for the stannate (Fig. 3b; similar results for the zirconate can be found in the literature^12^). This is consistent with the conventional understanding of proton–dopant association in acceptor-doped perovskites^15–17^. The H–Sn distribution increases slightly with increasing temperature, indicating that protons are slightly more randomly distributed, but a strong preference for H–Sc association remains at 527 °C. A high proton diffusivity is observed despite such a strong association, and this can be explained by the fast proton-conducting pathways formed along the three-dimensional network of ScO_6_ (Fig. 3c,d).

## Atomistic structure of BaSn_0.3_Sc_0.7_O_3−*δ*_

**Fig. 4:**
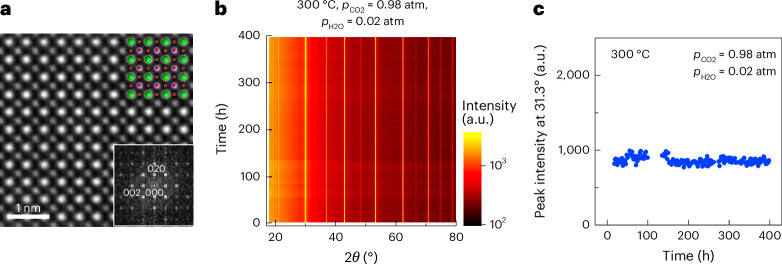
Structure and chemical stability of BaSn_0.3_Sc_0.7_O_3−*δ*_ electrolyte. **a**, HAADF-STEM image of dehydrated BaSn_0.3_Sc_0.7_O_3−*δ*_ electrolyte and digital diffractogram (inset at bottom right). **b**, Time course of in situ powder X-ray diffraction patterns for hydrated BaSn_0.3_Sc_0.7_O_3__−__*δ*_ under a concentrated and humidified CO_2_ stream. 2*θ*, the angle between the transmitted beam and reflected beam; *p*_CO2_, CO_2_ partial pressure. **c**, Amount of secondary BaSc_2_O_4_ plotted against exposure time to humidified CO_2_. The green, pink and red circles in **a** indicate the positions of the A sites (occupied by Ba), B sites (occupied by Sn and Sc) and O sites (occupied by O) in the perovskite-type structure, respectively. The time in **b** and **c** refers to the duration of the CO_2_-containing gas flow. Prior to the humidified CO_2_ flow, the powder sample was hydrated in an X-ray diffraction chamber for 9 h under a flowing humidified N_2_ stream with a water partial pressure (*p*_H2O_) of 0.02 atm. *θ* is the incident angle of X-ray. Colour in **b** represents the X-ray diffraction intensity. X-ray diffraction peak at 31.3° in **b** and **c** corresponds to the main peak of the BaSc_2_O_4_ phase. Assuming the degradation rate is proportional to the frequency of CO_2_ impacts on the material, and considering that the CO_2_ concentration during testing was 2,450 times that of ambient air (400 ppm), this accelerated stability test in **b** and **c** simulates over 100 years of exposure to ambient atmosphere at 300 °C. Source data

An analysis of the atomistic structure and chemical composition using transmission electron microscopy confirmed that the BaSn_0.3_Sc_0.7_O_3−*δ*_ electrolyte had a perovskite-type structure with a high Sc content. Electron diffraction patterns were acquired from one crystal grain of the dehydrated BaSn_0.3_Sc_0.7_O_3−*δ*_ electrolyte with two different electron incidence angles approximately 90° apart (Supplementary Fig. 21). All the Bragg reflections were reasonably indexed assuming a perovskite-type structure with the space group ${Pm}\bar{3}m$. The lattice parameter of 0.42 nm deduced from the electron diffraction pattern was consistent with the X-ray diffraction result of 0.4143 nm (Supplementary Fig. 1a). Both high-angle annular dark-field scanning transmission electron microscopy (HAADF-STEM; Fig. 4a and Supplementary Fig. 22) and energy-dispersive X-ray spectroscopy (EDS; Supplementary Fig. 23) imaged the sublattices making up the perovskite-type structure: that is, A sites occupied by Ba and B sites occupied by Sn and Sc, although the sublattice of O was unclear from these observations. The results showed neither cation ordering, expected in the double perovskite-type structure, nor oxygen-vacancy ordering in the brownmillerite-type structure. An analysis of the HAADF-STEM images revealed a negligible change in the intensity of the B sites, suggesting that the distribution of Sn and Sc at the B sites is random. An EDS analysis determined the atomic ratio of Ba/Sn/Sc to be 1:0.30:0.67 (Supplementary Fig 22c,f and Supplementary Table 2), indicating that Sc constitutes 69 at.% of the B-site cations (Supplementary Table 5). The results are in good agreement with the chemical analysis using ICP-OES, considering the 5 wt% BaSc_2_O_4_ (Supplementary Table 2).

## CO_2_ tolerance of BaSn_0.3_Sc_0.7_O_3−*δ*_ at 300 °C

An accelerated chemical stability test, conducted under harsh, humidified CO_2_ conditions at 300 °C using in situ X-ray diffractometry, confirmed the chemical stability of hydrated BaSn_0.3_Sc_0.7_O_3−*δ*_. The diffraction patterns for hydrated BaSn_0.3_Sc_0.7_O_3−*δ*_ powder did not vary at 300 °C under a flow of highly concentrated CO_2_ for 398 h (Fig. 4b and Supplementary Fig. 24). The amount of the secondary BaSc_2_O_4_ phase, shown by the peak intensity at 31.3°, did not increase during the test (Fig. 4c). These results show the chemical stability of the BaSn_0.3_Sc_0.7_O_3−*δ*_ powder, in agreement with the stability expected for the perovskite phases^34^; it is yet to be determined whether these results indicate thermodynamic or kinetic factors or both.

## Discussion

**Fig. 5:**
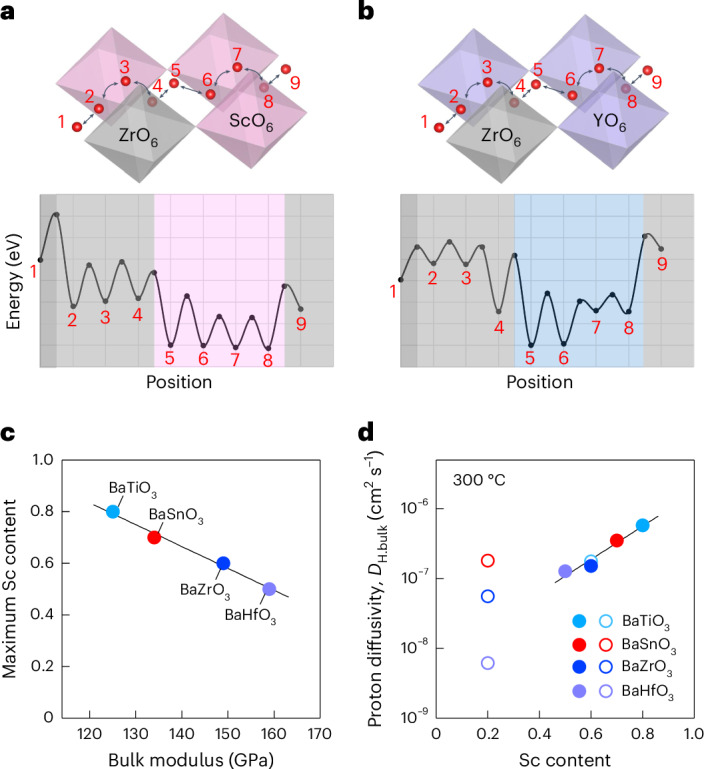
Fast conduction pathway for associated protons in heavily Sc-doped perovskite and descriptor for Sc solubility. **a**,**b**, Energy diagrams for proton conduction (shown at top) with heavy Sc (**a**) and Y (**b**) doping. **c**, Experimental maximum Sc content versus bulk modulus. **d**, Proton diffusivity versus Sc content. The motions between sites 2, 3 and 4 and between sites 6, 7 and 8 in **a** represent proton rotation about the Sc–O–Zr and Sc–O–Sc vectors, respectively, while the other motions represent proton hopping. The pink and blue regions in **a** and **b** show proton sites sandwiched with two Sc or Y dopants, respectively. The grey regions in **a** and **b** show proton sites sandwiched by one dopant and a Zr host, whereas the darker grey regions on the left correspond to a proton site sandwiched by two Zr host cations. The bulk moduli of the host oxides in **c** were adopted from the Materials Project^49^. Closed and open symbols in **d** denote data in the Sc solubility limit and solid solution, respectively. Source data

A distinct characteristic of Sc, particularly at higher concentrations, is its ability to create rapid diffusion pathways even when protons are strongly associated with acceptor dopants in the oxide. The measured proton diffusivity versus Sc content for various host cations (Figs. 1d and 5d) shows that the diffusivity at low Sc content (~20 at.%) is lower than that at higher Sc content (>50 at.%). This, in turn, indicates that proton trapping, which is unfavourable for proton diffusivity, is prevalent at lower Sc content but is mitigated at higher Sc content. Also notable is that the diffusivities exhibit a clear dependence on the host B-site cation at lower Sc content but converge to similar values at higher Sc content; this is a natural consequence of Sc becoming the major B-site cation under heavy doping. The microscopic picture responsible for this effect is evident from our molecular dynamics data for 60 at.% Sc-doped BaSnO_3_ (Supplementary Fig. 3c,d) and BaZrO_3_ (ref. ^12^). Sc doping increases the population of ScO_6_ octahedra, forming oxygen sites corner-shared by ScO_6_–BO_6_ and ScO_6_–ScO_6_ pairs. A further increase in Sc concentration creates a connected network of ScO_6_ octahedra, to which protons preferentially associate, as seen in our simulated data (Fig. 3c,d) and discussed in the literature^15–17^. Despite such a clear association, the calculated diffusivity is rather high as a result of proton migration along the ScO_6_ network without the need to completely detrap itself from the acceptor dopant.

To highlight the difference between Sc and Y dopants, we compare local migration barriers for Sc-doped and Y-doped BaZrO_3_, obtained from ab initio calculations on the dopant configuration shown in Fig. 5a,b. We find that associated protons along ScO_6_–ScO_6_ octahedra pairs from sites 5 to 8 (pink region in Fig. 5a) exhibit the lowest site energy (proton–dopant association), and the values are almost equal. Transitions between these sites encompass both proton rotation and hopping (Grotthuss mechanism) in the oxide. By contrast, Y-doped barium zirconate, which exhibits the concentration–conductivity trade-off^2,18^, shows a large difference of 0.18 eV in the site energies of protons at YO_6_–YO_6_ octahedra pairs (between sites 5 and 8 in Fig. 5b). Similar tendencies are observed in the ScO_6_–ZrO_6_ and YO_6_–ZrO_6_ pairs across sites 2 to 4 (grey regions in Fig. 5a,b). The overall activation energies for protons to move from one of the lowest energy sites to site 4 or 9 at the YO_6_–ZrO_6_ pair (0.42–0.51 eV) exceed those for Sc (approximately 0.3 eV). These results show the importance of forming an interconnected ScO_6_ octahedral network within the cubic perovskite structure for enhanced proton diffusion. Although heavy acceptor M-doping into perovskite oxides always leads to the formation of an MO_6_ octahedral network, creating a relatively flat energy landscape that mitigates proton trapping is a distinct benefit of using Sc as a dopant, while the cubic perovskite oxide retains structural stability under harsh chemical and fuel cell conditions.

Saito and Yashima^25^ have recently proposed starting from a hypothetical BaScO_2.5_ and adding Mo^6+^ or W^6+^ as donors to control the oxygen content for stabilization of the cubic state. BaScO_2.5_ exists in a brownmillerite structure at ambient pressure with limited proton conductivities^35^, but the cubic perovskite structure can be stabilized by applying 4 GPa (ref. ^36^) or by adding Mo^6+^ or W^6+^ into the Sc^3+^ site to introduce oxide ions; the resultant BaSc_0.8_Mo_0.2_O_3–*δ*_ (ref. ^25^) and BaSc_0.8_W_0.2_O_3–*δ*_ (ref. ^37^) adhere to the principle of an interconnected ScO_6_ octahedral network facilitating rapid proton diffusion in cubic perovskite.

A remaining issue is to characterize B-site cations that combine with Sc to stabilize Ba-based perovskite oxides with high Sc content. Although it becomes increasingly ambiguous whether BaScO_2.5_ or BaBO_3_ should be identified as the ‘host’ material at such high Sc content, considering the latter as the host turns out to be useful for our purpose; we find that the lattice softness (low bulk modulus) of ‘host’ BaBO_3_ correlates very strongly with high Sc ‘solubility’ (Fig. 5c). This experimental observation is also consistent with our ab initio calculations: the computed solution energy for Sc in the host oxides aligns with their maximum Sc content (Supplementary Fig. 25a) and decreases as the bulk modulus decreases (Supplementary Fig. 25b). The soft nature of the host lattice is also confirmed on a microscopic level by the lattice compression upon the formation of ${{\mathrm{v}}}_{{\rm{O}}}^{\bullet \bullet }$, which is linearly correlated with the bulk modulus (Supplementary Fig. 25c). This intrinsic lattice softness may reduce the energy increase associated with structural distortions induced by point defects, thereby facilitating Sc incorporation. A similar decreasing trend in solution energy for lower bulk modulus is also observed for Y (Supplementary Fig. 25d), indicating the general applicability of lattice softness as a descriptor for enhanced dopant content. Y doping of up to 50 at.% (ref. ^19^) with lower proton conductivities^20^ has been reported in BaZrO_3_, but this is not inconsistent with the above discussion; the solution energy is calculated for B-site substitution while experiments report partial substitution on the A site^38^ and/or phase separation^19^, which would lower the connectivity of the dopant network. We also considered the correlation between maximum Sc content and the ionic radius of the B-site cation in six-coordinate species, including Mo^6+^ and W^6+^, but the trend is not as clear (Supplementary Fig. 26).

The favourable impact of extremely heavy doping had been overlooked for almost half a century, since the first report of proton conduction in perovskite oxides^1^. Our work suggests that such heavy doping, enabled by the choice of host materials with ‘soft’ lattices, could open new avenues for high-performance materials for various solid-state devices.

## Methods

### Synthesis

BaSn_0.3_Sc_0.7_O_3−*δ*_, BaTi_0.2_Sc_0.8_O_3−*δ*_, BaTi_0.4_Sc_0.6_O_3−*δ*_, BaHf_0.5_Sc_0.5_O_3−*δ*_, BaSn_0.8_Sc_0.2_O_3−*δ*_ and BaHf_0.8_Sc_0.2_O_3−*δ*_ were synthesized using a solid-state reaction method and sintered at 1,600 °C for 12–24 h in dry air. The resulting relative densities of BaSn_0.3_Sc_0.7_O_3−*δ*_, BaTi_0.2_Sc_0.8_O_3−*δ*_, BaTi_0.4_Sc_0.6_O_3−*δ*_, BaHf_0.5_Sc_0.5_O_3−*δ*_, BaSn_0.8_Sc_0.2_O_3−*δ*_ and BaHf_0.8_Sc_0.2_O_3−*δ*_ pellets were 97%, 97%, 99%, 90%, 68% and 50%, respectively.

### Structural and chemical characterization

Crystal structure data were obtained from sintered pellets using an X-ray diffractometer (D2 Phaser, Bruker AXS) equipped with a Ni filter. The DIFFRAC.TOPAS software (v.5, Bruker AXS) was used for Rietveld refinements. The chemical composition of BaSn_0.3_Sc_0.7_O_3−*δ*_ was determined using ICP-OES at the Center of Advanced Instrumental Analysis, Toshiba Nanoanalysis Corporation.

### Thermogravimetry

The proton concentration of Sc-doped barium stannates, barium titanates and barium hafnates was evaluated using thermogravimetric analysis (STA449F3 Jupiter, NETZSCH). The proton concentration was determined in the temperature range 100–1,000 °C. The samples were first dehydrated at 1,000 °C for 2 h in dry Ar and subsequently exposed to a wet gas flow saturated with a water partial pressure of 0.02 atm. The proton concentration was calculated by assuming a weight gain from the dehydrated state to the hydrated state based on the hydration reaction ${{\mathrm{v}}}_{{\mathrm{O}}}^{\bullet \bullet }+{{\mathrm{O}}}_{{\mathrm{O}}}^{\times }+{{\mathrm{H}}}_{2}{\mathrm{O}}\leftrightarrow 2{\mathrm{O}}{{\mathrm{H}}}_{{\mathrm{O}}}^{\bullet }$. To reduce the buoyancy effect, each weight change measurement using thermogravimetric analysis was corrected by performing the measurement without the sample under the identical temperature and gas flow conditions.

### Transmission electron microscopy

Thin-foil specimens of a dehydrated BaSn_0.3_Sc_0.7_O_3−*δ*_ pellet, which were transparent to incident electrons, were prepared using a focused ion beam system with an acceleration voltage of 16 kV for shaping and 2 kV for thinning (at the final stage of polishing). In addition to polishing, electron backscatter diffraction and scanning electron microscopy analyses were carried out using a Helios 5 UX DualBeam (Thermo Fisher Scientific). Focused ion beam sample preparation was performed after dehydrating the BaSn_0.3_Sc_0.7_O_3−*δ*_ pellet by ramping up the temperature to 1,000 °C at a rate of 20 °C min^−1^ under 1 × 10^−10^ atm and maintaining the temperature at 1,000 °C for 1.5 h. Transmission electron microscopy, electron diffraction, HAADF-STEM and EDS were carried out using a JEM-ARM300F2 (JEOL) microscope at an acceleration voltage of 300 kV.

### Electrochemical characterization

Temperature-dependent electrical conductivities of sintered BaSn_0.3_Sc_0.7_O_3−*δ*_, BaSn_0.8_Sc_0.2_O_3−*δ*_, BaTi_0.2_Sc_0.8_O_3−*δ*_, BaTi_0.4_Sc_0.6_O_3−*δ*_, BaHf_0.5_Sc_0.5_O_3−*δ*_ and BaHf_0.8_Sc_0.2_O_3−*δ*_ pellets were measured by a.c. impedance spectroscopy. The pellets were polished to ~500 μm thickness and coated with 650 nm Ag films on both surfaces by d.c. sputtering (Sanyu Electron, SC-701HMC II). Silver meshes (Niraco Corporation) affixed with silver paste (Tanaka Kikinzoku Kogyo, TR-3025) acted as current collectors. Specimens were pre-annealed at 720 °C in dry Ar and then exposed to humidified Ar (*p*_H2O_ = 0.02 atm). The impedance spectra were acquired with an electrochemical station (BioLogic VSP-300) in the temperature range of 35–631 °C under the humidified atmosphere over a range from 10^−1^ Hz to 3 × 10^6^ Hz and fitted to an equivalent circuit of serial *RC* components, where *R* and *C* were in parallel, for the grain interior, grain boundary and electrode regions using EC-Lab software (v.11.33, Biologic Science Instruments). Here, *R* is the resistance from an ideal resistor with impedance *Z*_*R*_ = *R*, and *C* is a constant phase element with *Z*_*C*_ = (*Y*(*jω*)^*l*^)^−1^, where *j* is $\sqrt{-1}$, *ω* is the frequency and *Y* and *l* are constants with 0 ≤ *l* ≤ 1. H/D isotope effects were evaluated for BaSn_0.3_Sc_0.7_O_3−*δ*_ at 300 °C by switching between H_2_O-saturated and D_2_O-saturated Ar streams (*p*_H2O_ = *p*_D2O_ = 0.02 atm). The oxygen partial pressure dependence of proton conductivities in BaSn_0.3_Sc_0.7_O_3−*δ*_ was determined at 300 °C under *p*_H2O_ = 0.02 atm across the range from *p*_O2_ = 2.4 × 10^−22^ atm to 9.6 × 10^−1^ atm.

### In situ X-ray diffraction measurements under concentrated and humidified CO_2_ streams

To confirm the stability of BaSn_0.3_Sc_0.7_O_3−*δ*_ against CO_2_, high-temperature X-ray diffraction (D8 DISCOVER, Bruker AXS) was performed in a high-temperature chamber (XRK900, Anton Paar). Cu Kα radiation was used in the measurement under humidified CO_2_ (*p*_H2O_ = 0.02 atm, *p*_CO2_ = 0.98 atm) at 300 °C. Prior to exposure to humidified CO_2_, protons were introduced while maintaining the temperature at 300 °C in wet N_2_ (*p*_H2O_ = 0.02 atm) for 9 h. Note that the BaCO_3_ phase did not exist before exposure to the CO_2_ atmosphere. The diffraction patterns were continuously recorded in *θ*–2*θ* mode for 398 h.

### In situ FT-IR in a humidified atmosphere

Diffuse reflectance infrared Fourier transform spectroscopy (DRIFTS) was conducted on a Nicolet iS50 (Thermo Fisher Scientific) at 300 °C. Powders of BaSn_0.3_Sc_0.7_O_3__−__*δ*_, BaTi_0.2_Sc_0.8_O_3__−__*δ*_, BaSn_0.8_Sc_0.2_O_3__−__*δ*_ and BaTi_0.4_Sc_0.6_O_3__−__*δ*_ were first dehydrated at 1,000 °C for 1 h and subsequently introduced into the in situ chamber and heated to 300 °C under dry Ar. Spectra obtained in this dry atmosphere served as the reference. The gas flow was then switched to wet Ar (*p*_H2O_ = 0.02 atm), and spectra were collected up to 9 h.

### X-ray photoelectron spectroscopy

The Sn valence state in reduced BaSn_0.3_Sc_0.7_O_3−*δ*_ was analysed by X-ray photoelectron spectroscopy (AXIS-ULTRA, KRATOS Analytical). Measurements employed a monochromatic Al Kα X-ray source. Prior to measurement, the powder was reduced at 300 °C in flowing humidified H_2_ (partial pressure of H_2_ *p*_H2_ = 0.98 atm; *p*_H2O_ = 0.02 atm), corresponding to *p*_O2_ = 2 × 10^−47^ atm. Surface charging was done with an electron flood gun, and binding energy values were referenced to the C 1*s* peak at 285.0 eV.

### Electrolyte-supported protonic ceramic cells

A BaSn_0.3_Sc_0.7_O_3−*δ*_ disc pellet with a thickness of 0.3 mm was used as the electrolyte. Platinum paste (Tanaka Precious Metal, TR-7907) was applied on both sides of the disc as cathode and anode electrodes in a circular pattern with a diameter of 8 mm. The discs were then baked at 700 °C for 2 h in dry air, followed by a temperature reduction to 100 °C for 100 h in moist air. As the reference electrode, a Pt wire was attached to the side of the disc, while a Pt mesh attached to both electrodes using Ag paste (TR-3025) served as the current collector. After the Ag paste dried, 60 μl of 2 mol% cerium nitrate solution was dropped onto both electrodes. The samples were then fired at 300 °C for 2 h in humid air.

### Anode-supported protonic ceramic cells

Anode-supported proton ceramic single cells were fabricated using BaSn_0.3_Sc_0.7_O_3−*δ*_ electrolyte through cold-pressing and dip-coating. A mixture of 70 wt% Nano NiO (Kceracell), 30 wt% BaCe_0.7_Zr_0.1_Y_0.1_Yb_0.1_O_3−*δ*_ and 20 wt% starch was pressed at 100 MPa and pre-sintered at 900 °C for 2 h. The electrolyte film was dip-coated using 100 μl of a BaSn_0.3_Sc_0.7_O_3−*δ*_ slurry and then sintered at 1,300 °C for 5 h. PrBaCo_2_O_5+*δ*_ mixed with BaCe_0.7_Zr_0.1_Y_0.1_Yb_0.1_O_3−*δ*_ was brush-painted onto the electrolyte and sintered at 950 °C for 2 h. Ag and Pd were applied to the cathode and anode, respectively, as current collectors. The single cells were sealed in alumina tubes using glass sealant. The electrochemical measurements were made using a BioLogic VSP-300 with 2% H_2_O/H_2_ (20 sccm) at the anode and 2% H_2_O/air (100 sccm) at the cathode. The microstructure of single cells was observed using a micro-calorimeter field emission scanning electron microscope (TES + ULTRA55, Zeiss).

### Molecular dynamics simulations using a machine learning force field

The calculations were all performed in a 3 × 3 × 3 cubic perovskite supercell corresponding to 60 at.% Sc doping in BaSnO_3_. First, cation configurations corresponding to equilibrium at 1,900 K, which corresponds roughly to the sintering temperature, were obtained by replica exchange Monte Carlo calculations combined with a neural-network configuration energy model using our abICS framework^50,51^. The dry sintering conditions were simulated with a supercell containing 27 Ba, 16 Sc, 11 Sn and 73 O. Details of the calculation procedure and the neural-network model can be found in our previous work that studied 22 at.% Sc-doped BaZrO_3_ (ref. ^17^). The cation configuration is fixed to that determined by these Monte Carlo calculations, in subsequent molecular dynamics calculations using LAMMPS code^52^. We employed the Allegro model^53^ as the machine learning force field, and its training was performed in the fully hydrated system containing 27 Ba, 16 Sc, 11 Sn, 81 O and 16 H. The Supplementary Information contains details of the training procedure. In the final production run, deuterium was used instead of hydrogen to slow the local vibrations and enable accurate equation-of-motion integration with a time step of 1 fs. The diffusivities were calculated from 10 ns trajectories at 600–800 K and 100 ns trajectories at 500 K and 550 K. The calculated values are multiplied by $\sqrt{2}$ to compare the simulations using deuterium with experiments using protons.

### Proton migration barriers

The proton migration (hopping and rotation) barriers were calculated using the Vienna Ab initio Simulation Package for a 4 × 4 × 4 cubic perovskite supercell with 64 Ba, 61 Zr, 3 B (B = Sc, Y), 192 O and 1 H, where the B site dopants Sc and Y were placed as shown in Fig. 5a. Only the gamma point was used for k-point sampling. The plane-wave cut-off was set at 500 eV. A homogeneous background charge was introduced to satisfy charge neutrality considering formal ionic charges. The climbing-image nudged elastic band method^54^ was used to calculate minimum energy paths for the proton migration processes shown in Fig. 5a and obtain energy barriers.

### Dopant solution

The solution energy of the Sc and Y dopants was calculated for cubic BaSnO_3_, BaTiO_3_, BaZrO_3_ and BaHfO_3_. The trigonal (*R*3*m*), orthorhombic (*Amm*2) and tetragonal (*P*4*mm*) phases were also calculated for BaTiO_3_, considering its phase stability. The solution energy of dopant M for B-site cations was calculated using the following equation^55^:

4 $$\Delta {E}_{{\rm{sol}}}=\left\{{E}_{{\rm{def}}}^{\sup }\left({{\rm{M}}}_{{\rm{B}}}^{{\prime} }\right)+\frac{1}{2}{E}_{{\rm{def}}}^{\sup }\left({{\mathrm{v}}}_{{\rm{O}}}^{\bullet \bullet }\right)+{\mu }_{{\rm{B}}}+\frac{1}{2}{\mu }_{{\rm{O}}}\right\}-\,\left\{{\mu }_{{\rm{M}}}+\frac{3}{2}{E}_{{\rm{per}}}^{\sup }\right\},$$

where ${E}_{{\rm{def}}}^{\sup}({{\rm{M}}}_{{\rm{B}}}^{{\prime}})$ and ${E}_{{\rm{def}}}^{\sup}({{\mathrm{v}}}_{{\rm{O}}}^{\bullet \bullet})$ are the energies of a charged supercell of a perovskite compound containing a dopant M (Sc or Y) and an oxide-ion vacancy, respectively. The lattice constants for these supercells were fixed during structural optimization. ${E}_{{\rm{per}}}^{\sup }$ is the energy of a perfect perovskite supercell, and *μ*_B_, *μ*_O_ and *μ*_M_ are the chemical potentials for B, O and M, respectively. The Supplementary Information contains details of the computational conditions.

## Online content

Any methods, additional references, Nature Portfolio reporting summaries, source data, extended data, supplementary information, acknowledgements, peer review information; details of author contributions and competing interests; and statements of data and code availability are available at 10.1038/s41563-025-02311-w.

## Supplementary information

Supplementary Information Supplementary Notes 1 and 2, Tables 1–5 and Figs. 1–26.

Supplementary Data 1 Statistical source data for Supplementary Figs. 1–20 and 24–26.

Supplementary Data 2 Density functional theory structure data for Supplementary Fig. 25.

Supplementary Data 3 Animation of the proton hopping and rotation processes observed during molecular dynamics simulation.

## Source data

Source Data Fig. 1 Proton conductivity data plotted against lattice volume in Fig. 1a, bulk proton conductivity data plotted in Fig. 1b, proton concentration data plotted in Fig. 1c, proton diffusivity data plotted in Fig. 1d, apparent activation energy data for proton diffusivity plotted in Fig. 1e and proton diffusivity data plotted against proton concentration in Fig. 1f.

Source Data Fig. 2 Ionic conductivity data plotted in Fig. 2a, H–D isotope effect data plotted in Fig. 2b, proton conductivity data plotted against oxygen partial pressure in Fig. 2c and proton diffusivity data plotted in Fig. 2d.

Source Data Fig. 3 Proton diffusivity data plotted in Fig. 3a and radial distribution function data plotted in Fig. 3b.

Source Data Fig. 4 Amount of secondary BaSc_2_O_4_ plotted against exposure time to humidified CO_2_ in Fig. 4c.

Source Data Fig. 5 Data of energy diagrams for proton conduction with heavy Sc and Y doping plotted in Fig. 5a,b, experimental maximum Sc content plotted against bulk modulus in Fig. 5c and proton diffusivity data plotted against Sc content in Fig. 5d.

## Data Availability

All data supporting the findings of this study are available in the article and its Supplementary Information except for the molecular dynamics trajectories and training data/hyperparameters for the machine learning potential, which are available via the ISSP Data Repository at https://datarepo.mdcl.issp.u-tokyo.ac.jp/repo/54. Source data are provided with this paper.
