# Author Correction: Mitigating proton trapping in cubic perovskite oxides via ScO_6_ octahedral networks

**DOI:** 10.1038/s41563-026-02580-z

**Published:** 2026-03-18

**Authors:** Kota Tsujikawa, Junji Hyodo, Susumu Fujii, Kazuki Takahashi, Yuto Tomita, Nai Shi, Yasukazu Murakami, Shusuke Kasamatsu, Yoshihiro Yamazaki

**Affiliations:** 1 Platform of Inter-/Transdisciplinary Energy Research (Q-PIT), Kyushu University, Fukuoka, Japan; 2 INAMORI Frontier Research Center, Kyushu University, Fukuoka, Japan; 3 Department of Materials, Kyushu University, Fukuoka, Japan; 4 Center for Energy Systems Design (CESD), International Institute for Carbon-Neutral Energy Research (WPI-I2CNER), Kyushu University, Fukuoka, Japan; 5 Division of Materials and Manufacturing Science, Osaka University, Suita, Japan; 6 Nanostructures Research Laboratory, Japan Fine Ceramics Center, Nagoya, Japan; 7 Department of Science, Yamagata University, Yamagata, Japan; 8 The Ultramicroscopy Research Center, Kyushu University, Nishi-ku, Fukuoka, Japan; 9 Academic Assembly (Faculty of Science), Yamagata University, Yamagata, Japan

**Keywords:** Fuel cells, Solid-state chemistry

Correction to: *Nature Materials* 10.1038/s41563-025-02311-w, published online 8 August 2025.

In the version of the article initially published, the scale bar in Fig. 3d was labelled “Proton density (×10^−5^ Å^−3^)” and read (from top to bottom) “1.0, 0.5, 0” but should have been labelled “Proton density (×10^−1^ Å^−3^)” and have read (from top to bottom) “1.7, 0.7, 0”. In the Fig. 3 caption, “1 × 10^−6^ Å^−3^” has been corrected to “1.4 × 10^−2^ Å^−3^”.

