# Supplementary material for: Mitigating proton trapping in cubic perovskite oxides via ScO6 octahedral networks
Source: Nat Mater. 2025 Aug 8;24(12):1949–56. doi: 10.1038/s41563-025-02311-w (PMC12657236; doi:10.1038/s41563-025-02311-w)
Supplement: Supplementary file 1 — Supplementary Notes 1 and 2, Tables 1–5 and Figs. 1–26. [file 41563_2025_2311_MOESM1_ESM.pdf]

nature materials

Supplementary information https://doi.org/10.1038/s41563-025-02311-w

Mitigating proton trapping in cubic perovskite oxides via ScO_6 octahedral networks

In the format provided by the authors and unedited

Table of Contents

1. Supplementary Notes

S1: Defect chemistry in acceptor-doped perovskite oxides and their hydration behaviours

S2: Materials and methods

2. Supplementary Tables

Table S1: Structural parameters for dehydrated samples at room temperature

Table S2: Chemical compositions of dehydrated $BaSn_{0.3}Sc_{0.7}O_{3-\delta}$ perovskite

Table S3: Hydration enthalpies and entropies of perovskite oxides

Table S4: Electrochemical resistance of anode-supported cell and $BaSn_{0.3}Sc_{0.7}O_{3-\delta}$ film electrolyte

Table S5: B-site composition of various dehydrated perovskites ($BaSn_{0.3}Sc_{0.7}O_{3-\delta}$, $BaSn_{0.8}Sc_{0.2}O_{3-\delta}$, $BaTi_{0.2}Sc_{0.8}O_{3-\delta}$ and $BaTi_{0.4}Sc_{0.6}O_{3-\delta}$)

3. Supplementary Figures

Figure S1: XRD patterns of dehydrated perovskite powder samples

Figure S2: Lattice volume as a function of Sc content

Figure S3~S8: Nyquist plots under water partial pressure of 0.02 atm.

Figure S9: Bulk proton conductivities in Sc-doped barium stannates, titanates and hafnates.

Figure S10: Thermogravimetry results

Figure S11: Van 't Hoff plot for hydration reaction

Figure S12: Hydration thermodynamics in perovskite oxides

Figure S13: *In situ* FT-IR spectra

Figure S14: Proton diffusivities in Sc-doped barium stannates, titanates and hafnates

Figure S15: Total conductivities under humidified atmosphere

Figure S16: Microstructure of $BaSn_{0.3}Sc_{0.7}O_{3-\delta}$ pellet

Figure S17: $BaSn_{0.3}Sc_{0.7}O_{3-\delta}$ electrolyte-supported protonic ceramic fuel cell at 300°C

Figure S18: Chemical composition of $BaSn_{0.3}Sc_{0.7}O_{3-\delta}$ film electrolyte on anode-supported cell

Figure S19: Electrochemical performance and microstructure of anode-supported cell

Figure S20: X-ray photoelectron spectra

Figure S21: Identification of crystal phase by transmission electron microscopy (TEM).

Figure S22: Direct observations of atomic arrangement and chemical composition.

Figure S23: Sublattices comprising perovskite-type structure.

Figure S24: XRD of $BaSn_{0.3}Sc_{0.7}O_{3-\delta}$ before and after stability test (398 h under CO_2/H_2O)

Figure S25: Solution energy for Sc and lattice softness for Ba-based perovskite oxides

Figure S26: Maximum Sc content vs. ionic radius of B-site cation in six coordination

S1. Defect chemistry in acceptor-doped perovskite oxides and their hydration behaviors

The defect chemistry of acceptor-doped perovskite oxides for tetravalent *B* cation is understood generally as follows. The substitution of a trivalent dopant *M* (e.g., Y or Sc) at the tetravalent *B*-site (e.g., Zr, Sn, Ti) induces the formation of oxygen vacancies to preserve lattice electroneutrality:

$$M_2O_3 + 2B_B^{\times} + O_O^{\times} = v_O^{\bullet\bullet} + 2M_B^{\prime} + 2BO_2. \qquad \text{(S1)}$$

Despite variations in *B*-site cations (Sn, Ti, Zr), these reactions equivalently facilitate oxygen vacancy formation. Upon moisture exposure, the doped perovskite oxides absorb water, leading to the filling of vacancies with hydroxyl groups,

$$v_O^{\bullet\bullet} + O_O^{\times} + H_2O = 2OH_O^{\bullet}, \qquad \text{(S2)}$$

a process referred to as hydration. In the ideal solution limit, the equilibrium constants for hydration can be described as

$$K_{hyd} = (\Delta S_{hyd}^{\circ} / R)\ exp\ (-\Delta H_{hyd}^{\circ} / RT). \qquad \text{(S3)}$$

This encapsulates the defect chemistry and hydration mechanisms relevant to acceptor-doped perovskite oxides.

The incorporated protons are often strongly associated with the acceptor dopant [1],

$$M_B^{\prime} + OH_O^{\bullet} = \left(M_B^{\prime}\ OH_O^{\bullet}\right)^{\times} \qquad \text{(S4)}$$

Here, $\left(M_B^{\prime}\ OH_O^{\bullet}\right)^{\times}$ is the neutral complex of acceptor dopant and immobile associated protons.

S2. Materials and methods

Sample preparation

Dense pellets of $BaSn_{1-x}Sc_xO_{3-\delta}$ (x = 0.2 and 0.7 in nominal composition) were synthesized by a solid-state reaction. $BaCO_3$ (Wako Pure Chemical Industries, Ltd., 99.9%), SnO_2 (HIGH PURITY CHEMICALS, 99.9%), and Sc_2O_3 (HIGH PURITY CHEMICALS, 99.9%up) were used as the starting materials. The final calcination was done at 1100°C for 10 h in ambient air with a ramp rate of 200 $°C\cdot h^{-1}$. The precursor was ground by ball milling for 3 days using a zirconia medium in ethanol. The dried powder was pressed uniaxially, and the obtained discs were subsequently pressed with cold isostatic pressing at 300 MPa. The compacts were sintered at 1600°C for 12 h under dry air with a flow rate of 200 $ml\cdot min^{-1}$. During the final sintering process, the discs were

covered with powder with the same chemical composition to prevent the evaporation of the Ba element. The density of the prepared $BaSn_{0.3}Sc_{0.7}O_{3-\delta}$ pellet was 5.6 g·cm^{-3}, corresponding to 97% of the theoretical density estimated from the crystal structural analysis of the dehydrated sample.

Chemical and microstructural analyses

The chemical composition of cation species for an $x = 0.7$ sample was found to be $BaSn_{0.297}Sc_{0.701}O_{3-\delta}$ using inductively coupled plasma optical emission spectroscopy (ICP-OES, SPS-3520UV, Hitachi High-Technologies). The microstructure at the fracture surface of the sample was observed using scanning electron microscopy (SEM, TM4000Plus, HITACHI). The grain size distribution was obtained by counting 298 grains in an SEM image (**Figure S16**).

Crystal structure analysis

The crystal structure of 70-at% Sc-doped barium stannates was determined using powder X-ray diffraction with Cu-K_α radiation (XRD, D2Phaser, Bruker AXS). Dehydrated and hydrated samples were measured. The hydrated samples were used after an equilibrated proton concentration measurement by thermogravimetric analysis (TGA). Dehydrated samples were prepared by annealing the hydrated samples at 1000°C for 2 h under a dry Ar flow in the TGA equipment to monitor the negligible proton incorporation. The obtained patterns were fitted using the Rietveld method to extract the structural information using commercially available software (Topas5, Bruker AXS). The lattice constant, crystallite size, and atomic displacement factor, U, were variables in the Rietveld refinement.

Machine-learning force field (MLFF) and molecular dynamics simulations

Using the supercell corresponding to 60 at% Sc-doped in $BaSnO_3$, a 20 ps-trajectory at 1000 K was generated by *ab initio* molecular dynamics (MD) calculations using VASP[2,3] code. A plane wave cutoff of 400 eV was employed with Brillouin zone sampling at the Γ-point only. Structures were then extracted from the trajectory and recalculated using a plane wave cutoff of 550 eV to provide high-accuracy energy and force data for the training. An initial MLFF was trained on this data and used to run MD calculations for 10 ns. A relearning cycle was employed, where structures from the MLFF MD trajectory were recalculated using VASP and added to the training data set to iteratively improve the MLFF accuracy. The final training (validation) mean absolute errors for the interatomic forces were 0.07 (0.10) eV/Å for the training (validation) set with 1324 (234) samples.

The final MD calculations were performed using the MLFF, and proton diffusivities were calculated from the trajectories using MDAnalysis python library[4-7]. The temperatures are controlled by the Nosé thermostat with a damping parameter of 0.1 ps. 204 structure samples were extracted from the final trajectory and recalculated using VASP to evaluate the actual MLFF mean absolute error in the production run, which yielded an acceptable value of 0.05 eV/Å.

Dopant solution

Ab initio calculations were performed using the exchange correlation functional of PBEsol.[8] The plane-wave energy cutoff was set as high as 550 eV to accurately determine the lattice constants. Supercells with cell lengths larger than 10 Å (3 × 3 × 3 supercells for cubic perovskites) were used for calculating the energies $E_{\mathrm{def}}^{\mathrm{sup}}(\mathrm{M}_{\mathrm{B}}')$, $E_{\mathrm{def}}^{\mathrm{sup}}(v_{\mathrm{O}}^{\cdot\cdot})$, and $E_{\mathrm{per}}^{\mathrm{sup}}$ in Eq. (1) of the main manuscript. Monkhorst–Pack *k*-point meshes of 3 × 3 × 2 and 2 × 2 × 2 were employed for trigonal $BaTiO_3$ and for the other structures, respectively. The chemical potential terms in Eq. (1) were calculated by deriving the most energetically favorable solid solution reaction of M dopant with competing phases in a computed phase diagram using pymatgen.[9] The competing phases were taken from the Materials Project database and their energies were calculated under the same conditions for the charged defects except for *k*-point sampling with a spacing of 0.3 Å^{-1}. The energies of charged defects, $E_{\mathrm{def}}^{\mathrm{sup}}(\mathrm{M}_{\mathrm{B}}')$ and $E_{\mathrm{def}}^{\mathrm{sup}}(v_{\mathrm{O}}^{\cdot\cdot})$, were corrected by the extended Freysoldt–Neugebauer–Van de Walle scheme[10,11] using dielectric constants derived by density functional perturbation theory.[12,13] The volume change upon introducing an oxide-ion vacancy (formation volume) was calculated by subtracting the volume of the perfect supercell from the volume of the charged supercell with an oxide-ion vacancy, which was re-optimized allowing for changes in cell length and shape.

Table S1. Structural parameters for dehydrated $BaSn_{0.3}Sc_{0.7}O_{3-\delta}$, $BaTi_{0.2}Sc_{0.8}O_{3-\delta}$, $BaSn_{0.8}Sc_{0.2}O_{3-\delta}$, $BaTi_{0.4}Sc_{0.6}O_{3-\delta}$, $BaHf_{0.5}Sc_{0.5}O_{3-\delta}$ and $BaHf_{0.8}Sc_{0.2}O_{3-\delta}$ determined at room temperature. For $BaSn_{1-x}Sc_xO_{3-\delta}$ (x = 0.2 and 0.7) and $BaHf_{1-x}Sc_xO_{3-\delta}$ (x = 0.2 and 0.5), the variables in the Rietveld refinement were the lattice constant, crystallite size, occupancy, g, and atomic displacement factor, U, for perovskite as well as the lattice constant and crystallite size for $BaSc_2O_4$. For $BaTi_{0.2}Sc_{0.8}O_{3-\delta}$ the variables in the Rietveld refinement were the lattice constant, crystallite size, atomic displacement factor, U, for perovskite as well as the lattice constant and crystallite size for $BaSc_2O_4$. The chemical compositions determined by STEM-EDS for $BaSn_{0.3}Sc_{0.7}O_{3-\delta}$ and by SEM-EDS for the others were used as initial values for g. For $BaSn_{1-x}Sc_xO_{3-\delta}$ (x = 0.2 and 0.7) and $BaHf_{1-x}Sc_xO_{3-\delta}$ (x = 0.2 and 0.5), the total occupancies for B-sites were fixed at one. It gave the occupancy for $BaSn_{0.3}Sc_{0.7}O_{3-\delta}$ at 0.293 Sn and 0.707 Sc, and for $BaHf_{0.5}Sc_{0.5}O_{3-\delta}$ at 0.479 Hf and 0.521 Sc, respectively, after the refinements. The occupancies for the titanates were fixed at the values obtained from EDS measurements due to resolution issues. The g for O sites was determined based on the charge neutrality of the sample.

$BaSn_{0.3}Sc_{0.7}O_{3-\delta}$, R_{wp}: 8.53 %, R_{exp}: 5.47 %, GOF (= R_{wp}/R_{exp}): 1.56.

$BaSn_{0.3}Sc_{0.7}O_3$ perovskite (94.9(3) wt%)

Atom	Site	g	x	y	z	U/Å^2
Ba	1*b*	1.000(11)	0.5	0.5	0.5	0.0114(2)
Sn	1*a*	0.293(11)	0	0	0	0.0086(5)
Sc	1*a*	0.707(11)	=*x*(Sn)	=*y*(Sn)	=*z*(Sn)	=*U*(Sn)
O	3*d*	0.8822(19)	0.5	0	0	0.0167(18)

Space group: $Pm\bar{3}m$, a = 4.143314(16) Å, crystallite size: 173.2(11) nm,

$BaSc_2O_4$ (5.1(3) wt%)

Atom	Site	g (fixed)	x (refined)	y (refined)	z (refined)	U/Å^2 (fixed)
Ba1	1a	1	0	0.035(6)	0.25	0.012665
Ba2	2a	1	0.338(9)	0.035(6)	0.1482(18)	0.012665
Sc1	1b	1	0.16(3)	0.53(5)	0.182(6)	0.012665
Sc2	2b	1	0.09(2)	0.72(3)	-0.001(6)	0.012665
Sc3	3b	1	0(2)	0.02(5)	0.095(5)	0.012665
O1	1c	1	0	0.55(14)	0.25	0.012665

O2	2c	1	0.17(6)	0.53(12)	0.223(13)	0.012665
O3	3c	1	0.06(6)	0.80(9)	0.11(2)	0.012665
O4	4c	1	0.08(6)	0.28(8)	0.118(11)	0.012665
O5	5c	1	0.33(5)	0.55(10)	0.118(11)	0.012665
O6	6c	1	0.26(16)	0.3(4)	0(18)	0.012665
O7	7c	1	0.0000	0.5000	0.0000	0.012665

Space group: $C12/c1$. a = 10.126(10) Å, b = 5.761(5) Å, c = 20.718(11) Å, crystallite size: 23(4) nm.

$BaSn_{0.8}Sc_{0.2}O_{3-\delta}$, R_{wp}: 10.38 %, R_{exp}: 5.38 %, GOF (= R_{wp}/R_{exp}): 1.93.

Atom	Site	g	x	y	z	U/Å^2
Ba	1b	0.97(2)	0.5	0.5	0.5	0.0066(5)
Sn	1a	0.81(3)	0	0	0	0.0048(6)
Sc	1a	0.19(3)	=x(Sn)	=y(Sn)	=z(Sn)	=U(Sn)
O	3d	0.969(5)	0.5	0	0	0.013(3)

Space group: $Pm\bar{3}m$, a = 4.12650(2) Å, crystallite size: 141.2(8) nm

$BaTi_{0.2}Sc_{0.8}O_{3-\delta}$, R_{wp}: 10.18 %, R_{exp}: 5.51 %, GOF (= R_{wp}/R_{exp}): 1.85.

$BaTi_{0.2}Sc_{0.8}O_3$ perovskite (97.6(1) wt%)

Atom	Site	g	x	y	z	U/Å^2
Ba	1b	1.000(4)	0.5	0.5	0.5	0.019(2)
Ti	1a	0.24	0	0	0	0.0260(8)
Sc	1a	0.76	=x(Ti)	=y(Ti)	=z(Ti)	=U(Ti)
O	3d	0.8733	0.5	0	0	0.029(1)

Space group: $Pm\bar{3}m$, a = 4.160695(19) Å, crystallite size: 202.6(12) nm

$BaSc_2O_4$ (2.3(1) wt%)

Atom	Site	g (fixed)	x (fixed)	y (fixed)	z (fixed)	U/Å^2 (fixed)
Ba1	1a	1	0	0.109	0.25	0.006333
Ba2	2a	1	0.3344	0.109	0.1385	0.006333
Sc1	1b	1	0.1662	0.608	0.1969	0.006333
Sc2	2b	1	0.0028	0.619	0.053	0.006333

Sc3	3b	1	0	0.117	0.0697	0.006333
O1	1c	1	0	0.57	0.25	0.006333
O2	2c	1	0.264	0.373	0.248	0.006333
O3	3c	1	0.065	0.839	0.123	0.006333
O4	4c	1	0.103	0.356	0.121	0.006333
O5	5c	1	0.336	0.653	0.121	0.006333
O6	6c	1	0.153	0.943	0.0025	0.006333
O7	7c	1	0.0000	0.5000	0.0000	0.006333

Space group: $C12/c1$. a = 10.047(3) Å, b = 5.7921(15) Å, c = 20.186(3) Å, crystallite size: 89(14) nm.

$BaTi_{0.4}Sc_{0.6}O_{3-\delta}$, R_{wp}: 9.38 %, R_{exp}: 5.42 %, GOF (= R_{wp}/R_{exp}): 1.73.

Atom	site	g	x	y	z	U/Å^2
Ba	1b	1.000(3)	0.5	0.5	0.5	0.0196(2)
Ti	1a	0.43	0	0	0	0.0300(8)
Sc	1a	0.57	=x(Ti)	=y(Ti)	=z(Ti)	=U(Ti)
O	3d	0.905	0.5	0	0	0.032(1)

Space group: $Pm\bar{3}m$, a = 4.14945(3) Å, crystallite size: 178(4) nm

$BaHf_{0.5}Sc_{0.5}O_{3-\delta}$, R_{wp}: 6.20 %, R_{exp}: 4.23 %, GOF (= R_{wp}/R_{exp}): 1.47.

$BaHf_{0.5}Sc_{0.5}O_3$ perovskite (94.7(3) wt%)

Atom	site	g	x	y	z	U/Å^2
Ba	1b	1.000	0.5	0.5	0.5	0.0174(5)
Hf	1a	0.479(3)	0	0	0	0.0036(4)
Sc	1a	0.521(3)	=x(Hf)	=y(Hf)	=z(Hf)	=U(Hf)
O	3d	0.9132(5)	0.5	0	0	0.0182(11)

Space group: $Pm\bar{3}m$, a = 4.163553(17) Å, crystallite size: 113.0(5) nm

$BaSc_2O_4$ (5.3(3) wt%)

Atom	Site	g (fixed)	x (fixed)	y (fixed)	z (fixed)	U/Å^2 (fixed)
Ba1	1a	1	0	0.049(6)	0.25	0.012665
Ba2	2a	1	0.375(4)	0.049(6)	0.153(3)	0.012665
Sc1	1b	1	0.185(14)	0.53(3)	0.163(7)	0.012665

Sc2	2b	1	0.086(13)	0.72(2)	0.007(6)	0.012665
Sc3	3b	1	0.008(14)	0.09(3)	0.091(7)	0.012665
O1	1c	1	0	0.49(9)	0.25	0.012665
O2	2c	1	0.21(3)	0.47(7)	0.239(18)	0.012665
O3	3c	1	0.06(6)	1.13(9)	0.11(2)	0.012665
O4	4c	1	0.07(5)	0.05(7)	0.136(12)	0.012665
O5	5c	1	0.23(4)	0.56(7)	0.136(12)	0.012665
O6	6c	1	0.2(5)	0.3(7)	0.00(13)	0.012665
O7	7c	1	0.0000	0.0000	0.0000	0.012665

Space group: $C12/c1$. a = 10.082(7) Å, b = 5.672(6) Å, c = 20.885(18) Å, crystallite size: 17.8(15) nm.

$BaHf_{0.8}Sc_{0.2}O_{3-\delta}$, R_{wp}: 6.28 %, R_{exp}: 3.89%, GOF (= R_{wp}/R_{exp}): 1.61.

Atom	site	g	x	y	z	U/Å^2
Ba	1b	1.000	0.5	0.5	0.5	0.0100(4)
Hf	1a	0.867(3)	0	0	0	0.0065(4)
Sc	1a	0.133(3)	=x(Hf)	=y(Hf)	=z(Hf)	=U(Hf)
O	3d	0.9779(5)	0.5	0	0	0.0150(11)

Space group: $Pm\bar{3}m$, a = 4.17105(3) Å, crystallite size: 68.8(7) nm

Table S2. Chemical compositions of dehydrated $BaSn_{0.3}Sc_{0.7}O_{3-\delta}$ perovskite determined using ICP-OES and STEM-EDS. The chemical compositions determined using STEM-EDS were normalized by the Ba content.

	Method	Ba	Sn	Sc	Amount of perovskite / wt%
Nominal	-	1	0.3	0.7	100
Actual	ICP-OES	1.004 ± 0.001	0.296 ± 0.002	0.698 ± 0.001	100
Actual	ICP-OES	1.000± 0.001[a]	0.311 ± 0.002[a]	0.695 ± 0.001[a]	94.87[a]
Actual	STEM-EDS	1	0.30 ± 0.02	0.67 ± 0.03	100

a: Cation contents of perovskite were determined assuming the existence of 5.13-wt% $BaSc_2O_4$ secondary phase shown in **Table S1**.

Table S3. Hydration enthalpies and entropies in Sc-doped barium stannates, titanates and hafnates. The equilibrium constants for the hydration reaction (K_{hyd}) were determined in the temperature range indicated, considering the presence of inactive oxygen vacancies for hydration.[14,15] $v_{O.inactive}$ represents oxygen vacancies that are not hydrated under these conditions.

Composition	Abbreviation	Temperature (℃)	$\Delta H_{hyd.exp}$ (kJ mol^{-1})	$\Delta S_{hyd.exp}$ (J K^{-1} mol^{-1})	[$v_{O.inactive}$]	Reference
$BaSn_{0.3}Sc_{0.7}O_{3-\delta}$	BSS70	417 ~ 912	−125±1	−134±1	0.022	this work
$BaSn_{0.8}Sc_{0.2}O_{3-\delta}$	BSS20	315 ~ 914	−131±3	−169±3	0.023	this work
$BaTi_{0.2}Sc_{0.8}O_{3-\delta}$	BTS80	518 ~ 914	−99±5	−116±5	0.032	this work
$BaTi_{0.4}Sc_{0.6}O_{3-\delta}$	BTS60	416 ~ 913	−71±2	−109±3	0.111	this work
$BaHf_{0.5}Sc_{0.5}O_{3-\delta}$	BHS50	416 ~ 912	−112±2	−123±3	0.020	this work
$BaHf_{0.8}Sc_{0.2}O_{3-\delta}$	BHS20	417 ~914	−80±2	−97±2	0.032	this work
$BaZr_{0.4}Sc_{0.6}O_{3-\delta}$	BZS60	450 ~ 1000	−121±2	−117±2	0.023	[16]
$BaZr_{0.8}Sc_{0.2}O_{3-\delta}$	BZS20	450 ~ 1000	−104±1	−96±1	0.011	[16]
$BaZr_{0.9}Y_{0.1}O_{3-\delta}$	BZY10	~500 ~ ~900	−79.4	−88.8	-	[17]
$BaZr_{0.8}Y_{0.2}O_{3-\delta}$	BZY20	~550 ~ ~900	−93.3	−103.2	-	[17]
$BaZr_{0.7}Y_{0.3}O_{3-\delta}$	BZY30	492 ~ 885	−91±16	−124±17	-	[18]
$BaZr_{0.6}Y_{0.4}O_{3-\delta}$	BZY40	490 ~ 887	−80±10	−108±13	-	[18]

Table S4. Electrochemical resistance of anode-supported cell and $BaSn_{0.3}Sc_{0.7}O_{3-\delta}$ film electrolyte. $R_{DC.cell}$ refers to the resistance of a fuel cell determined from the slope of the *I-V* curves in **Figure S19a**. $R_{electrolyte.cell}$ and $R_{electrode.cell}$ represent the total resistance of the electrolyte and electrode, respectively, obtained from AC impedance spectra fitted to an equivalent circuit consisting of three serial *RC* components. $R_{AC.cell}$ is the sum of $R_{electrolyte.cell}$ and $R_{electrode.cell}$, indicating the total cell resistance determined by AC impedance. $R_{total.BSS70}$ represents the total resistance of $BaSn_{0.3}Sc_{0.7}O_{3-\delta}$, converted from **Figure 2a**. $R_{electrolyte.cell}R_{total\text{-}BSS70}^{-1}$ shows that the resistance of the film electrolyte in the cell is two to three times larger than that of the $BaSn_{0.3}Sc_{0.7}O_{3-\delta}$ pellet in **Figure 2a**, probably due to Ba deficiency and Ni incorporation in the electrolyte film (**Figure S18**). $R_{electrolyte.cell}R_{DC.cell}^{-1}$ at 300°C shows that the performance of the fuel cell is mainly restricted by the electrode (95%) rather than the electrolyte (5%).

Temperature / °C	$R_{DC.cell}$ / Ωcm^2	$R_{electrolyte.cell}$ / Ωcm^2	$R_{electrode.cell}$ / Ωcm^2	$R_{AC.cell}$ / Ωcm^2	$R_{total.BSS70}$* / Ωcm^2	$R_{electrolyte.cell}R_{total\text{-}BSS70}^{-1}$	$R_{electrolyte.cell}R_{DC.cell}^{-1}$ / %
500	4.5	1.4	2.6	4.0	0.91 (498.9°C)	1.5	31
450	7.5	1.9	7.1	9.0	0.87 (444.9°C)	2.2	25
400	15.6	2.6	32.4	35.0	0.80 (395.9°C)	3.3	17
350	37.2	3.9	-	-	1.12 (354.1°C)	3.5	10
300	101.3	5.1	-	-	1.82 (299.6°C)	2.8	5

* Calculated from the measured total proton conductivities in **Figure 2a**. The electrolyte thickness of 18 μm is determined from **Figure S18c** and the effective electrode area of 0.096 cm^2.

Table S5. B-site contents for dehydrated $BaSn_{0.3}Sc_{0.7}O_{3-\delta}$, $BaSn_{0.8}Sc_{0.2}O_{3-\delta}$, $BaTi_{0.2}Sc_{0.8}O_{3-\delta}$ and $BaTi_{0.4}Sc_{0.6}O_{3-\delta}$ perovskites. The composition of $BaSn_{0.3}Sc_{0.7}O_{3-\delta}$ perovskite was determined using STEM-EDS (details given in **Figures S22** and **S23**) while those of $BaSn_{0.8}Sc_{0.2}O_{3-\delta}$, $BaTi_{0.2}Sc_{0.8}O_{3-\delta}$ and $BaTi_{0.4}Sc_{0.6}O_{3-\delta}$ perovskites were determined using SEM-EDS.

Materials	Sn / at%	Sc / at%
$BaSn_{0.3}Sc_{0.7}O_{3-\delta}$ (dehydrated)	31	69
$BaSn_{0.8}Sc_{0.2}O_{3-\delta}$ (dehydrated)	83	17

Materials	Ti / at%	Sc / at%
$BaTi_{0.2}Sc_{0.8}O_{3-\delta}$ (dehydrated)	24	76
$BaTi_{0.4}Sc_{0.6}O_{3-\delta}$ (dehydrated)	43	57

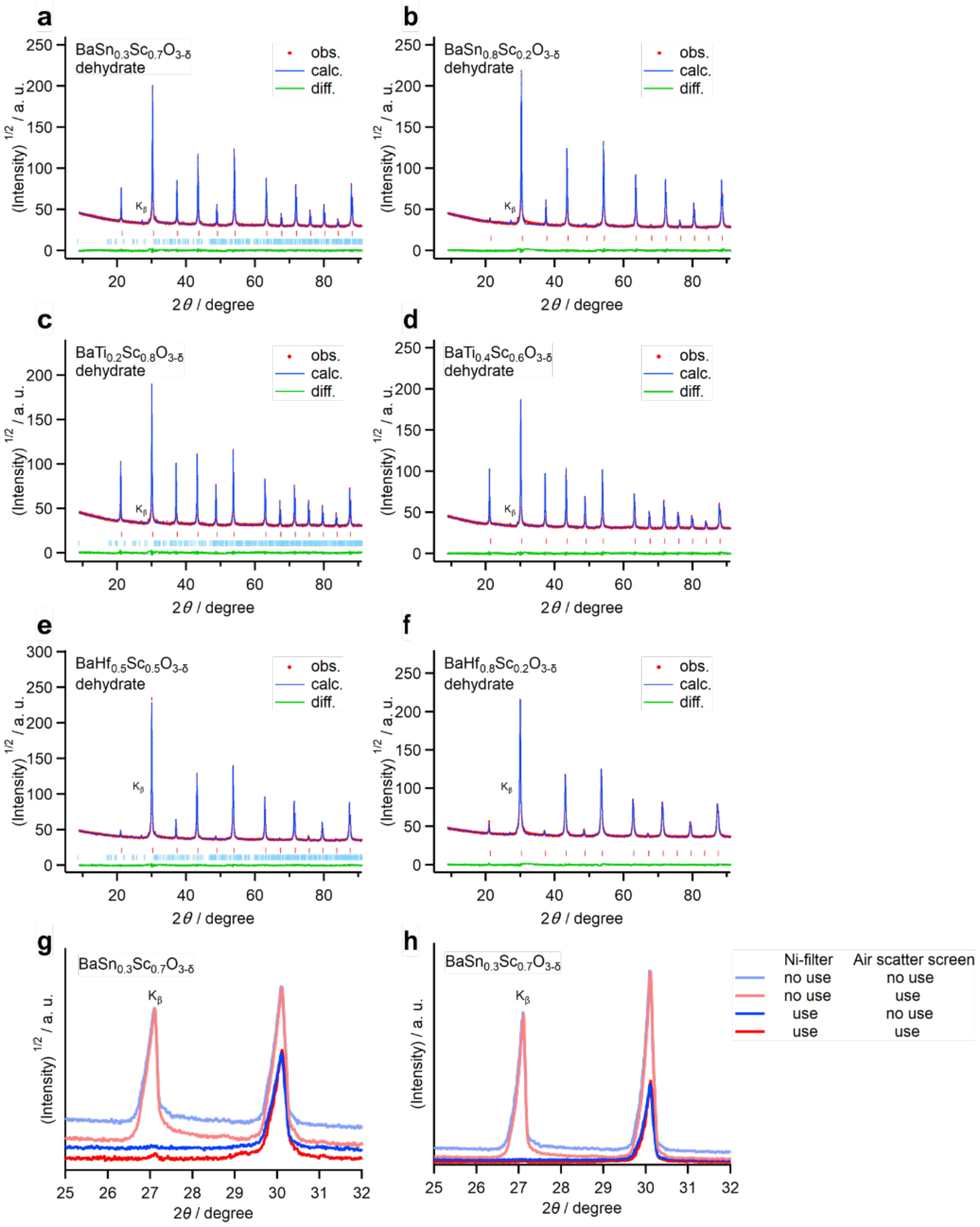

Figure S1. Powder X-ray diffraction patterns for dehydrated perovskites. **a**, $BaSn_{0.3}Sc_{0.7}O_{3-\delta}$, **b**, $BaTi_{0.2}Sc_{0.8}O_{3-\delta}$, **c**, $BaSn_{0.8}Sc_{0.2}O_{3-\delta}$, and **d**, $BaTi_{0.4}Sc_{0.6}O_{3-\delta}$, **e**, $BaHf_{0.5}Sc_{0.5}O_{3-\delta}$ and **f**, $BaHf_{0.8}Sc_{0.2}O_{3-\delta}$, **g**, enlarged figure for $BaSn_{0.3}Sc_{0.7}O_{3-\delta}$, **h**, enlarged figure with linear y-scale for $BaSn_{0.3}Sc_{0.7}O_{3-\delta}$. The vertical lines show peaks for the phases considered in the Rietveld refinement, in which the blue patterns and green lines represent the calculated patterns and the differences between the calculated and measured intensity, respectively. The small peak at 27.3° corresponds to the *110* diffraction from Cu-K_β radiation. The sample was treated in vacuum at 1000°C for 1 hour and then quenched to room temperature.

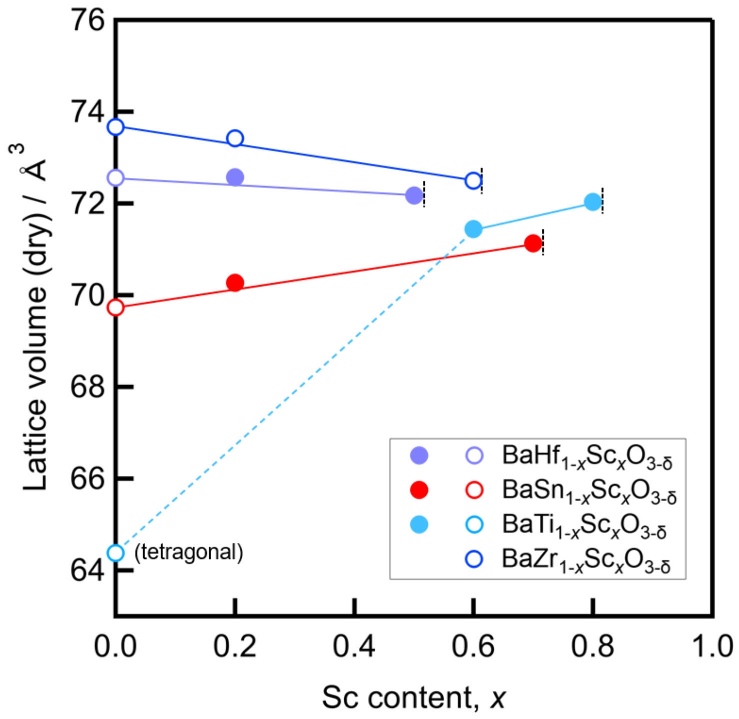

Figure S2. Lattice volume against Sc content. The data for $BaZr_{0.4}Sc_{0.6}O_{3-\delta}$ and $BaZr_{0.8}Sc_{0.2}O_{3-\delta}$, $BaZrO_3$, $BaTiO_3$, $BaSnO_3$ and $BaHfO_3$ are taken from the literature.[16,19-22] The vertical dashed lines indicate the solubility limit for Sc. With the exception of undoped $BaTiO_3$, all exhibit a cubic perovskite structure. Monotonic increase or decrease in lattice volume up to 70, 80, 60 and 50 at% were observed for barium stannates, titanates, zirconates[16] and hafnates, respectively, indicative of Sc solubility limits. The increase/decrease rate vs. Sc content can be explained by the ionic radius of the substitutional Sc^{3+} dopant (0.745 Å) compared to the parent *B* cation (0.72 Å for Zr^{4+}, 0.69 Å for Sn^{4+} and 0.605 Å for Ti^{4+} in six coordination)[23] and the contribution of oxygen vacancies with a negative formation volume[24]. It is noted that barium titanate exhibits a non-linear increase with Sc doping due to a structural transition from tetragonal in an undoped sample to cubic in the 60- and 80-at% Sc-doped samples.

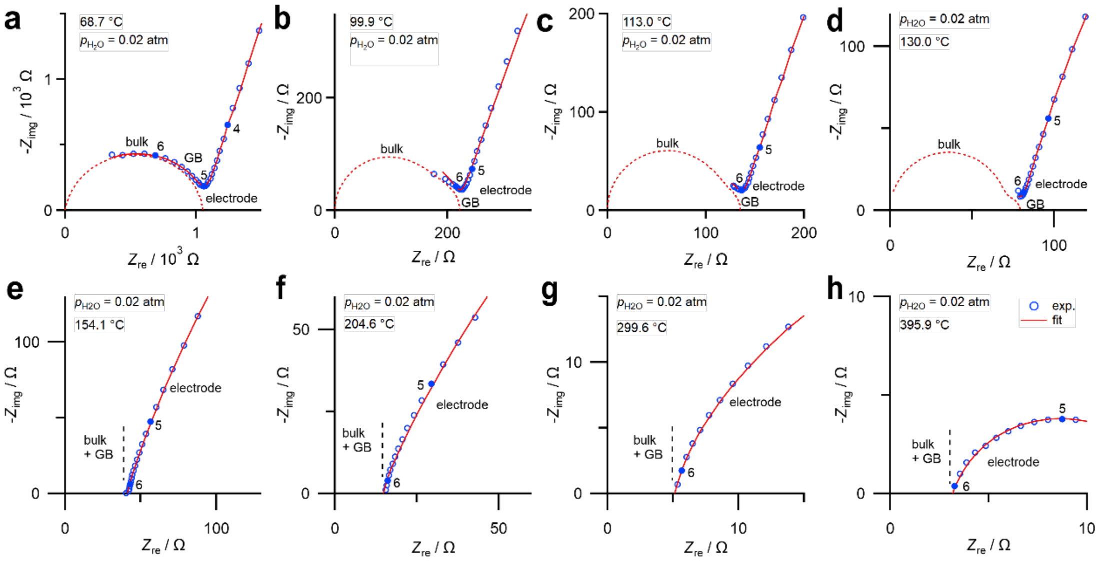

Figure S3. **Nyquist plots for $BaSn_{0.3}Sc_{0.7}O_{3-\delta}$ obtained under water partial pressure of 0.02 atm.** The number beside each blue closed marker corresponds to the exponent, n, of the base ten, 10^n, in frequency. The red dashed lines in **a** to **d** represent the calculated values expanded from the fitting parameters for the bulk and/or grain boundaries. The black dashed lines in **e** to **h** show the total resistance obtained from the fitting.

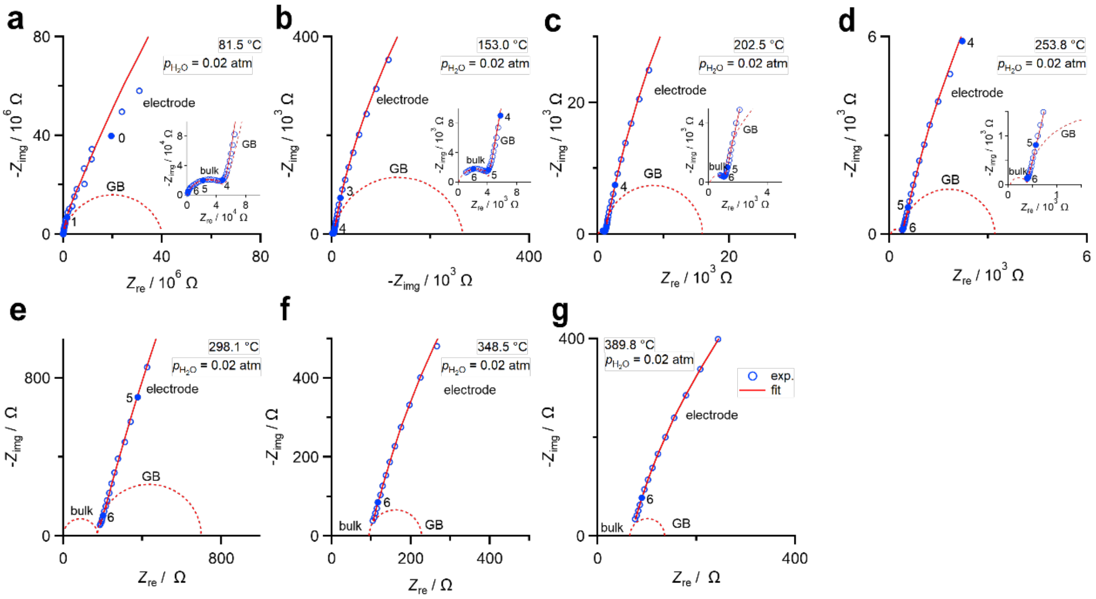

Figure S4. **Nyquist plots for $BaSn_{0.8}Sc_{0.2}O_{3-\delta}$ obtained under water partial pressure of 0.02 atm.** The number beside each blue closed marker corresponds to the exponent, n, of the base ten, 10^n, in frequency. The red dashed lines represent the calculated values expanded from the fitting parameters for the bulk and/or grain boundaries.

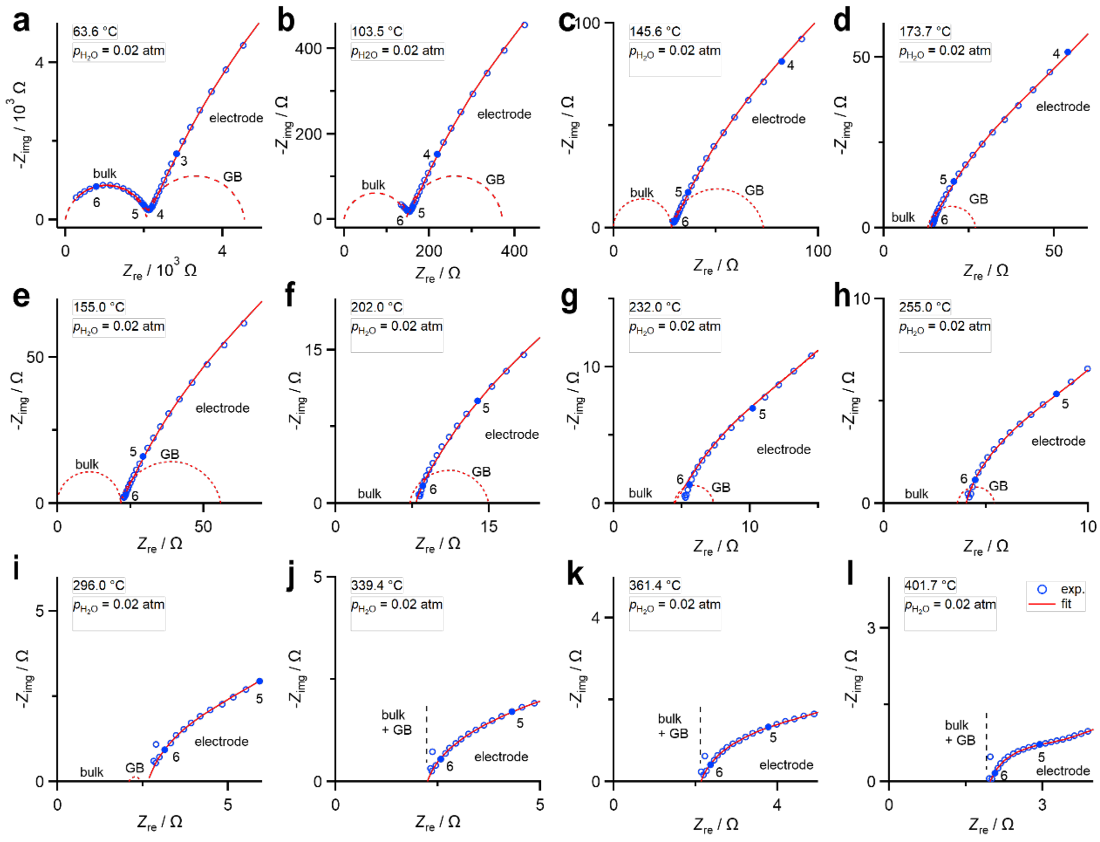

Figure S5. **Nyquist plots for $BaTi_{0.2}Sc_{0.8}O_{3-\delta}$ obtained under water partial pressure of 0.02 atm.** The number beside each blue closed marker corresponds to the exponent, *n*, of the base ten, 10^n, in frequency. The red dashed lines in **a** to **i** represent the calculated values expanded from the fitting parameters for the bulk and/or grain boundaries. The black dashed lines in **j** to **i** show the total resistance obtained from the fitting.

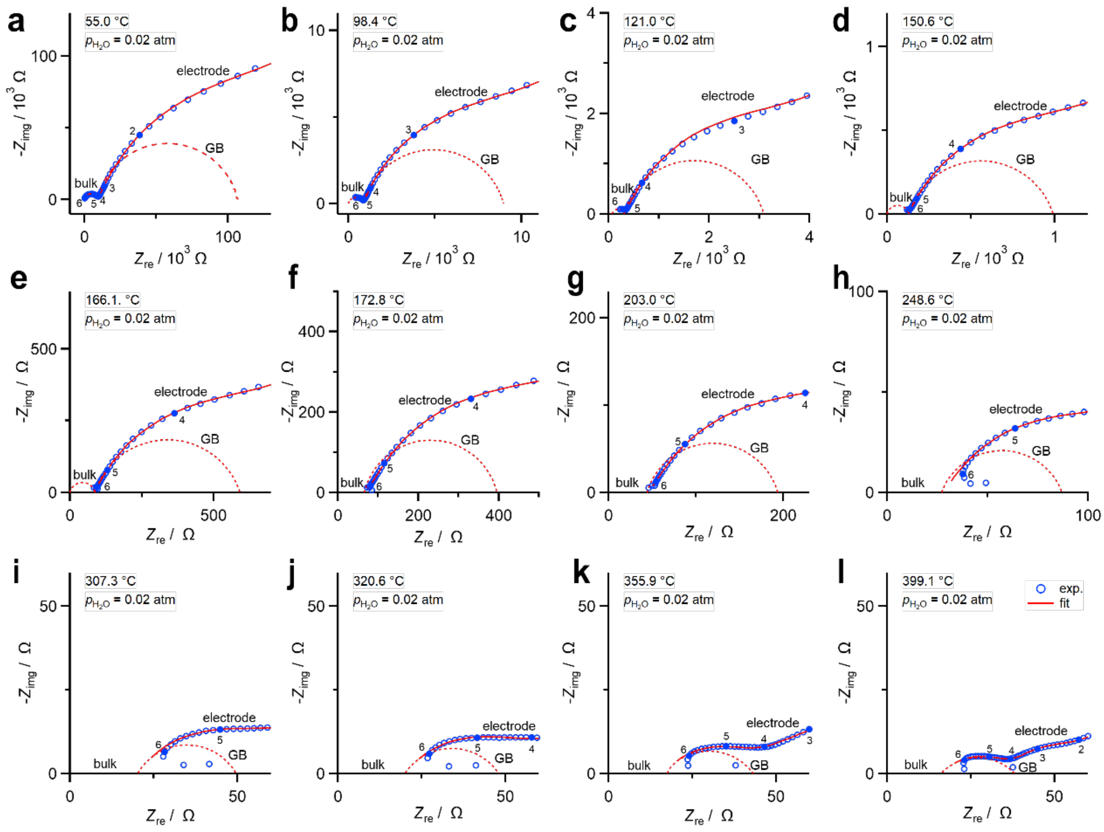

Figure S6. Nyquist plots for $BaTi_{0.4}Sc_{0.6}O_{3-\delta}$ obtained under water partial pressure of 0.02 atm. The number beside each blue closed marker corresponds to the exponent, *n*, of the base ten, 10^n, in frequency. The red dashed lines represent the calculated values expanded from the fitting parameters for the bulk and/or grain boundaries.

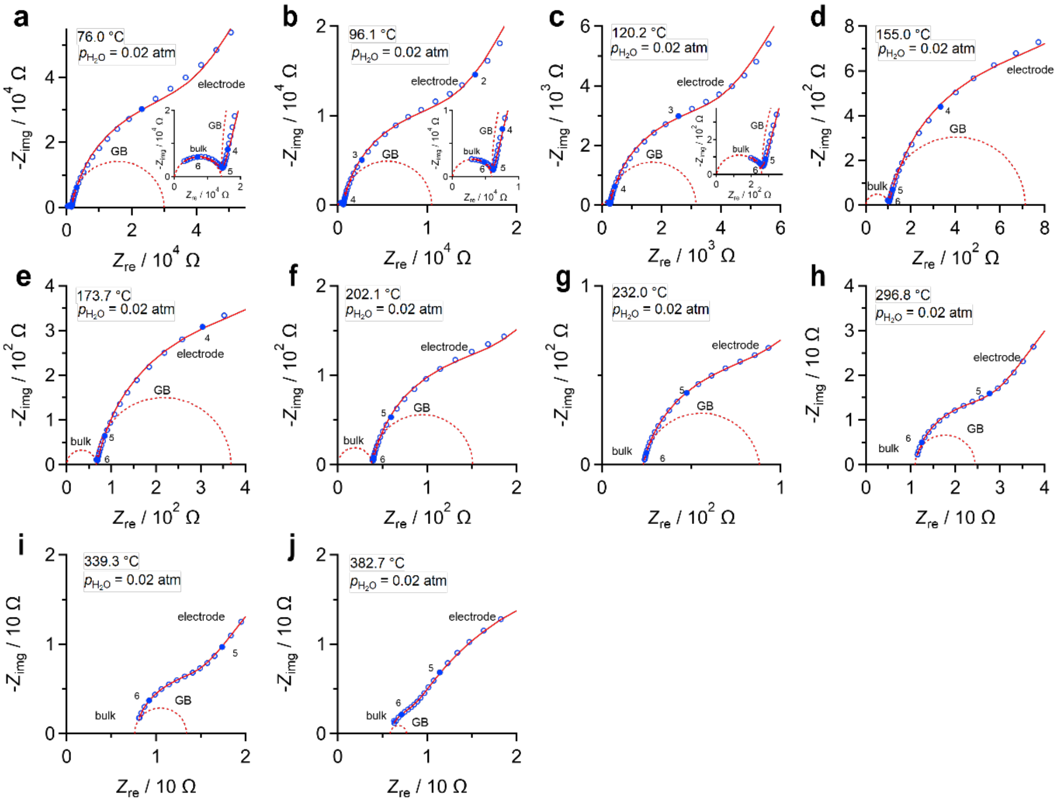

Figure S7. Nyquist plots for $BaHf_{0.5}Sc_{0.5}O_{3-\delta}$ obtained under water partial pressure of 0.02 atm. The number beside each blue closed marker corresponds to the exponent, n, of the base ten, 10^n, in frequency. The red dashed lines represent the calculated values expanded from the fitting parameters for the bulk and/or grain boundaries.

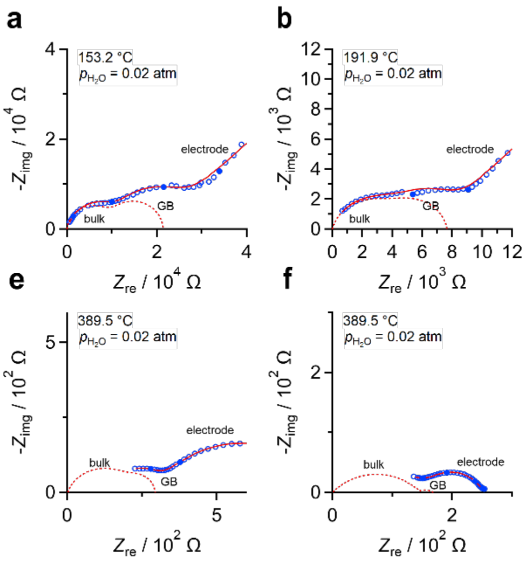

Figure S8. Nyquist plots for $BaHf_{0.8}Sc_{0.2}O_{3-\delta}$ obtained under water partial pressure of 0.02 atm. The number beside each blue closed marker corresponds to the exponent, *n*, of the base ten, 10^n, in frequency. The red dashed lines represent the calculated values expanded from the fitting parameters for the bulk and/or grain boundaries.

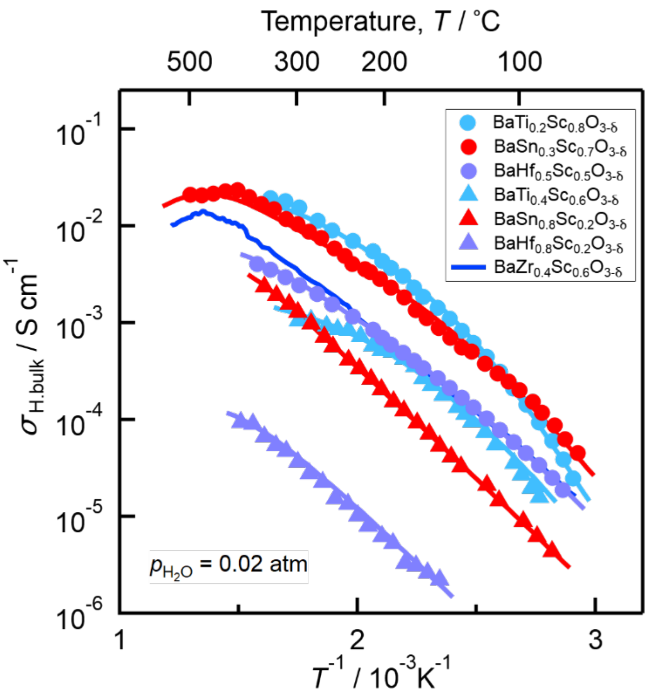

Figure S9. Bulk proton conductivities in Sc-doped barium stannates, titanates and hafnates. The data for $BaZr_{0.4}Sc_{0.6}O_{3-\delta}$ are taken from the literature.[16]

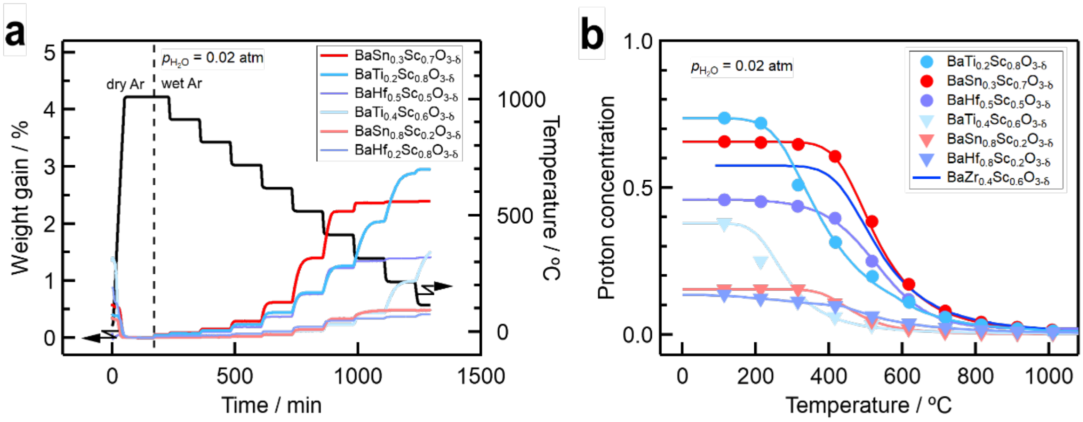

Figure S10. Thermogravimetry results for Sc-doped $BaSnO_{3-\delta}$, $BaTiO_{3-\delta}$ and $BaHfO_{3-\delta}$. **a**, Raw data and **b**, proton concentration against temperature. The data for $BaZr_{0.4}Sc_{0.6}O_{3-\delta}$ are taken from the literature.[16] All samples exhibited proton concentration increase with decreasing temperature.

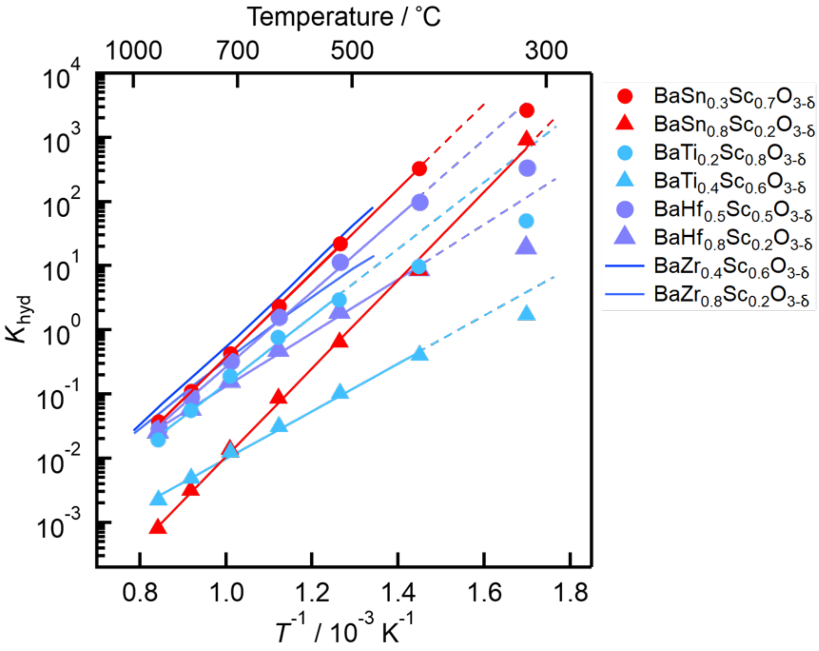

Figure S11. Van 't Hoff plot for hydration reaction in 20- and 70-at% Sc-doped barium stannates, 60- and 80-at% Sc-doped barium titanates and 20- and 50-at% Sc-doped barium hafnates. The data for $BaZr_{0.4}Sc_{0.6}O_{3-δ}$ and $BaZr_{0.8}Sc_{0.2}O_{3-δ}$ are taken from the literature.[16]

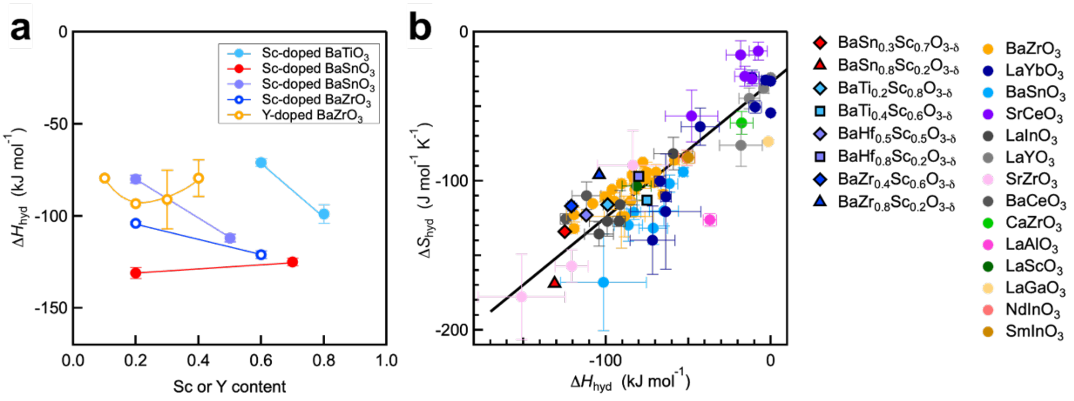

Figure S12. Hydration thermodynamics in perovskite oxides. a, Hydration enthalpy against Sc content in barium-based perovskites and **b**, hydration enthalpy vs. hydration entropy in perovskite oxides.[25] Error bars in **a** represent the standard error of the slope obtained from the least-squares fits of the van 't Hoff plots, based on 6, 7, 5, 6, 6 and 6 data points for $BaSn_{0.3}Sc_{0.7}O_{3-\delta}$, $BaSn_{0.8}Sc_{0.2}O_{3-\delta}$, $BaTi_{0.2}Sc_{0.8}O_{3-\delta}$, $BaTi_{0.4}Sc_{0.6}O_{3-\delta}$, $BaHf_{0.5}Sc_{0.5}O_{3-\delta}$ and $BaHf_{0.8}Sc_{0.2}O_{3-\delta}$, respectively. The data for Sc-[16] and Y[17,18]-doped barium zirconates in **a** are reproduced from the literature. Hydration enthalpies and their associated errors for $BaZr_{0.7}Y_{0.3}O_{3-\delta}$ and $BaZr_{0.6}Y_{0.4}O_{3-\delta}$[18] were recalculated using 5 data points within 500-900°C temperature range. The remaining data in **b** were reproduced from reference[25].

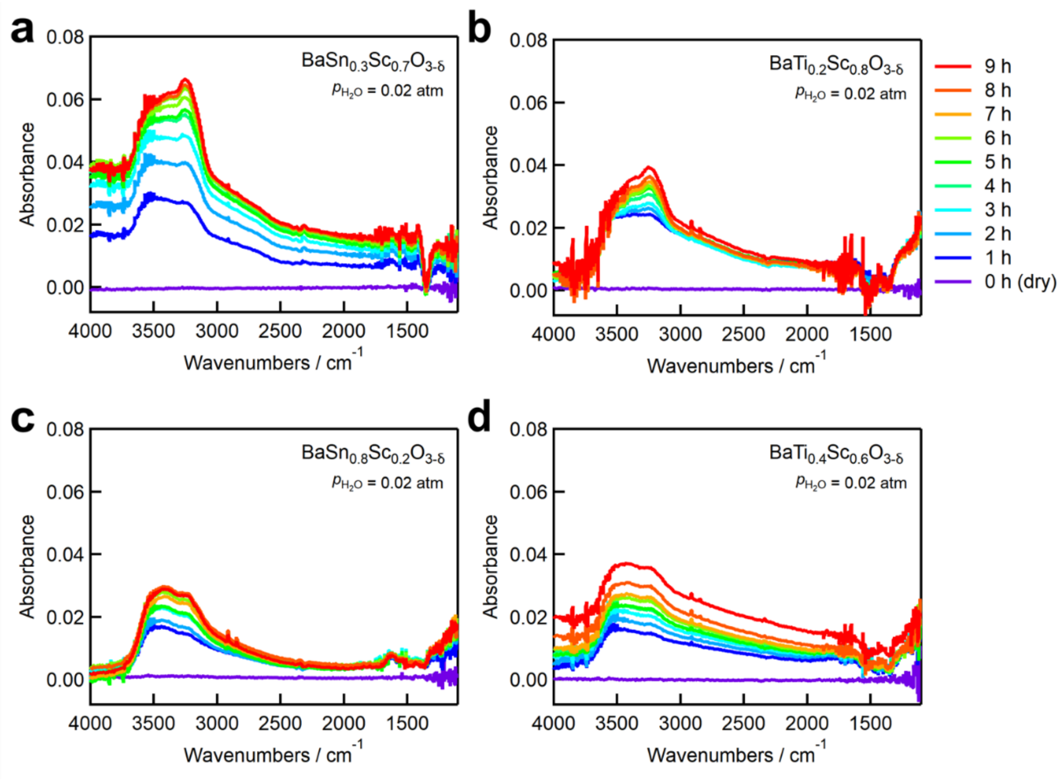

Figure S13. ***In situ*** **FT-IR spectra under flowing humidified Ar at 300 °C. a) $BaSn_{0.3}Sc_{0.7}O_{3-δ}$, b) $BaTi_{0.2}Sc_{0.8}O_{3-δ}$, c) $BaSn_{0.8}Sc_{0.2}O_{3-δ}$ and d) $BaTi_{0.4}Sc_{0.6}O_{3-δ}$.** When the atmosphere was switched from dry argon to humidified argon with a water partial pressure of 0.02 atm at 300°C for 20% and 70% Sc-doped barium stannates, as well as 60% and 70% Sc-doped barium titanates, FT-IR spectra exhibited a broad peak in the 3000-3700 cm^{-1} range, indicative of O-H stretching vibrations associated with hydroxyl groups, as observed in 64% Sc-doped $BaZrO_3$[26]. The continuous increase in the intensity of this peak over time demonstrates the accumulation of hydroxyl groups. These results provide direct evidence of hydration reaction and its defect chemistry outlined in eq. (2) for Sc-doped barium stannates and titanates.

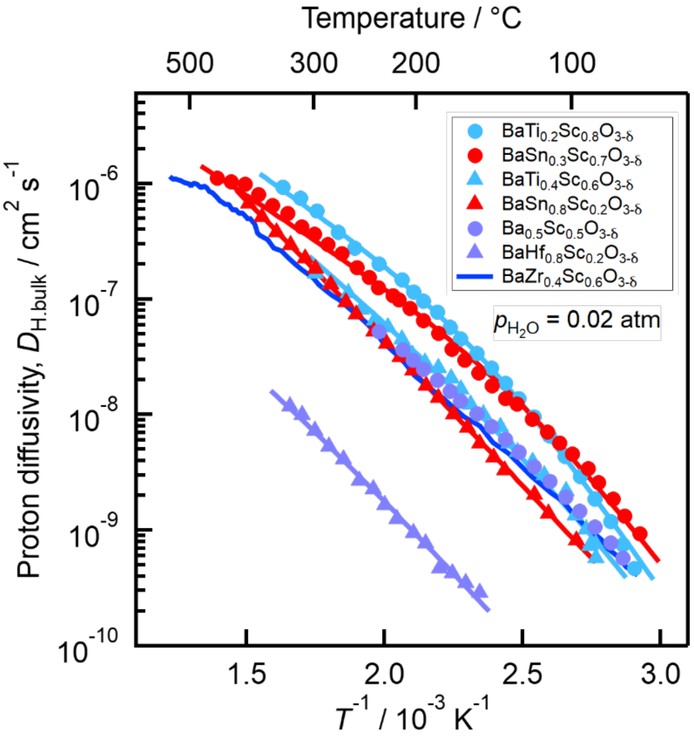

Figure S14. Proton diffusivities in Sc-doped barium stannates, titanates and hafnates. The data for $BaZr_{0.4}Sc_{0.6}O_{3-\delta}$ are taken from the literature.[16]

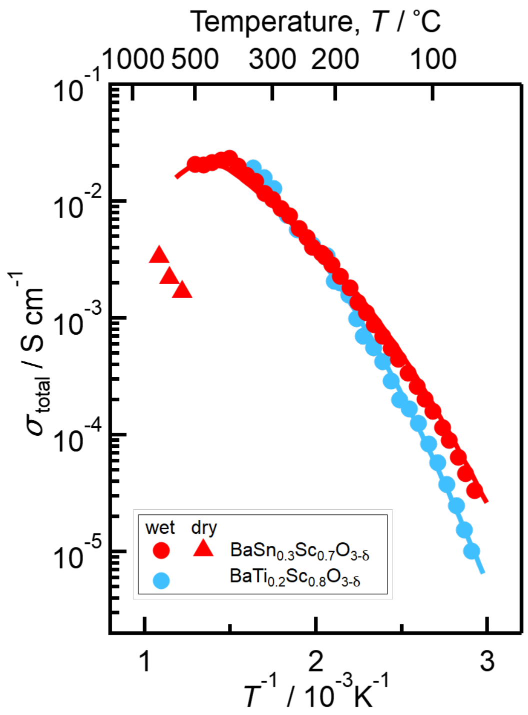

Figure 15. Total conductivities of $BaSn_{0.3}Sc_{0.7}O_{3-\delta}$ and $BaTi_{0.2}Sc_{0.8}O_{3-\delta}$ under humidified atmosphere. The experiments were conducted at the water partial pressures of 2×10^{-2} atm (wet) and 2.5×10^{-4} atm (dry). Under wet conditions at 300°C, total proton conductivities of 1.0×10^{-2} Scm^{-1} for $BaSn_{0.3}Sc_{0.7}O_{3-\delta}$ and 1.3×10^{-2} Scm^{-1} for $BaTi_{0.2}Sc_{0.8}O_{3-\delta}$ were obtained. In contrast, under dry conditions, the total conductivities for $BaSn_{0.3}Sc_{0.7}O_{3-\delta}$ decreases by an order of magnitude.

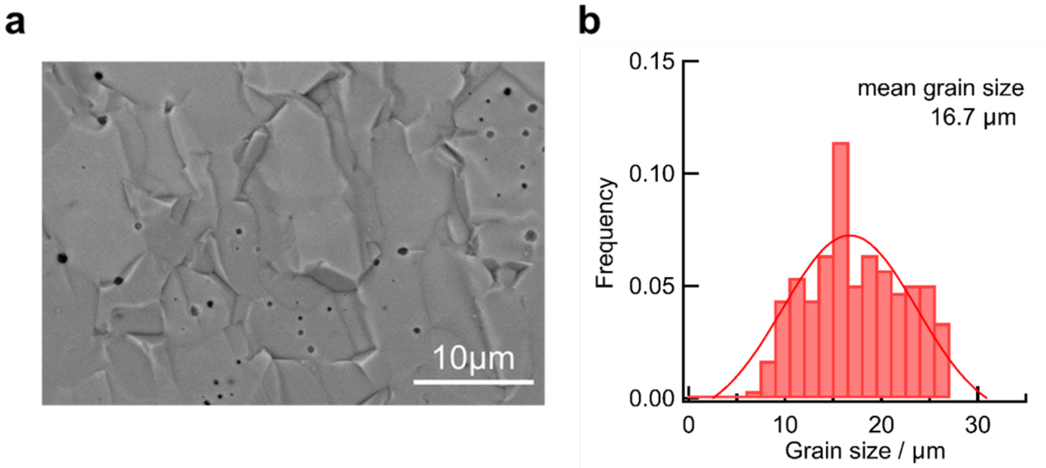

Figure S16. Microstructure of $BaSn_{0.3}Sc_{0.7}O_{3-\delta}$ pellet. a, Cross-sectional scanning electron microscopy image and **b**, grain size distribution. The mean diameter of grains was determined to be 16.7 ± 0.2 μm, with a standard deviation of 4.8 ± 0.3 μm, from an examination of 298 grains in the SEM image.

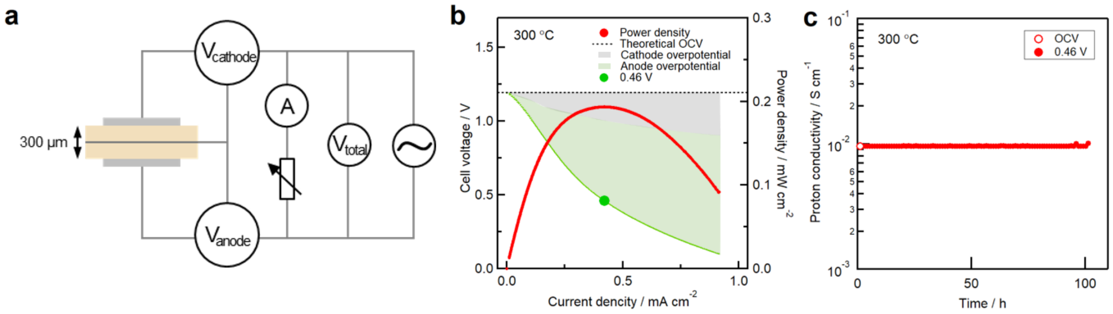

Figure S17. $BaSn_{0.3}Sc_{0.7}O_{3-\delta}$ electrolyte-supported protonic ceramic fuel cell at 300°C. a, Cell configuration of H_2-2-vol% H_2O, Pt-Ce | $BaSn_{0.3}Sc_{0.7}O_{3-\delta}$ | Pt-Ce, 21-vol% O_2-2-vol% H_2O-Ar. **b**, Open-circuit voltage and *I-V* curve. **c**, proton conductivity of electrolyte under fuel cell operation at 0.46 V (peak power density) and 300°C. The thickness of the $BaSc_{0.7}Sn_{0.3}O_{3-\delta}$ electrolyte for the electrolyte-supported cells was 300 μm. The ohmic loss within the electrolyte during fuel cell operation at 0.46 V was minimal, recorded at only 0.0004 V, which is equivalent to a proton conductivity of 0.01 $S{\cdot}cm^{-1}$ over a duration of 100 h in **c**. The output power density was 0.19 $mW{\cdot}cm^{-2}$ at 300°C. The cathodic and anodic overpotentials at a current density of 0.42 $mA{\cdot}cm^{-2}$ were 0.19 V and 0.54 V, respectively. These results show that $BaSn_{0.3}Sc_{0.7}O_{3-\delta}$ serves as an effective electrolyte in intermediate-temperature proton ceramic fuel cells.

a

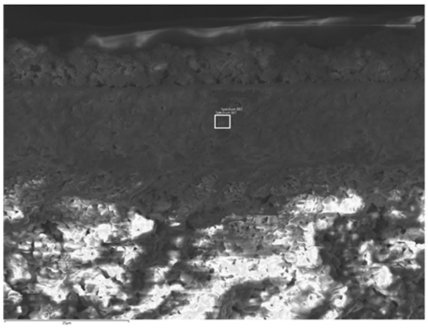

b

Cation	Atomic ratio
Ba	0.84
Sn	0.23
Sc	0.57
Ni	0.20

Figure S18. Chemical composition of $BaSn_{0.3}Sc_{0.7}O_{3-\delta}$ film electrolyte on anode-supported cell. a, Cross-sectional SEM image. **b**, Atomic ratio of cations determined from energy dispersive X-ray spectra collected from square region in **a** assuming stoichiometric B-site content. Ni incorporation is detrimental to the proton conductivity of an electrolyte[27] and the performance of proton ceramic fuel cells.[28]

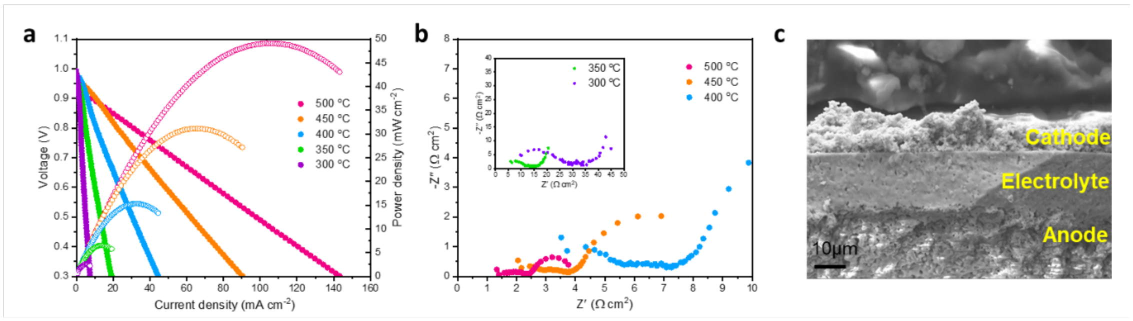

Figure S19. Electrochemical performance and microstructure of anode-supported cell. a, I-V-P curves at 300–500°C. **b**, Electrochemical impedance spectra under open-circuit voltages. **c**, Cross-section SEM image of fuel cell near film electrolyte. The fuel cell is composed of H_2-2-vol% H_2O, Ni-$BaCe_{0.7}Zr_{0.1}Y_{0.1}Yb_{0.1}O_{3-\delta}$ | $BaSn_{0.3}Sc_{0.7}O_{3-\delta}$ | $PrBaCo_2O_{5+\delta}$-$BaCe_{0.7}Zr_{0.1}Y_{0.1}Yb_{0.1}O_{3-\delta}$, air-2-vol% H_2O. The electrolyte thickness was found to be approximately 18 μm in **c**.

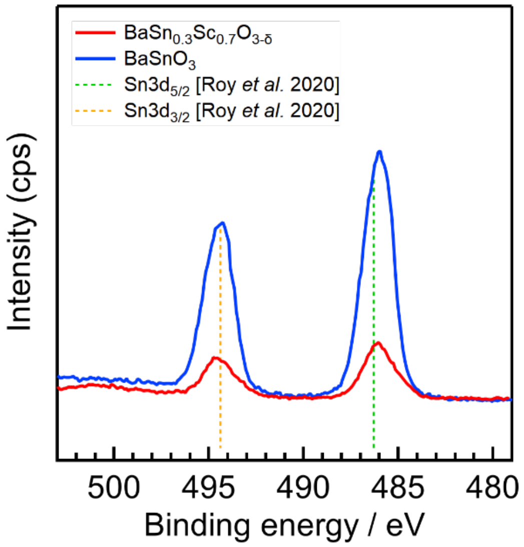

Figure S20. X-ray photoelectron spectra of reduced $BaSn_{0.3}Sc_{0.7}O_{3-\delta}$ electrolyte. The $BaSn_{0.3}Sc_{0.7}O_{3-\delta}$ powder was reduced under flowing H_2-2-vol% H_2O at 300°C, equivalent to $p_{O_2} = 2\times10^{-47}$ atm whereas the $BaSnO_3$ sample was sintered at 1600°C for 12 h in dry air. The dashed lines show the energies for $Sn3d_{5/2}$ and $Sn3d_{3/2}$, assigned to Sn^{4+} in $BaSnO_3$ sintered at 850°C in air.[29] The binding energy was calibrated using the reference peak of C_{1s} (285.0 eV).

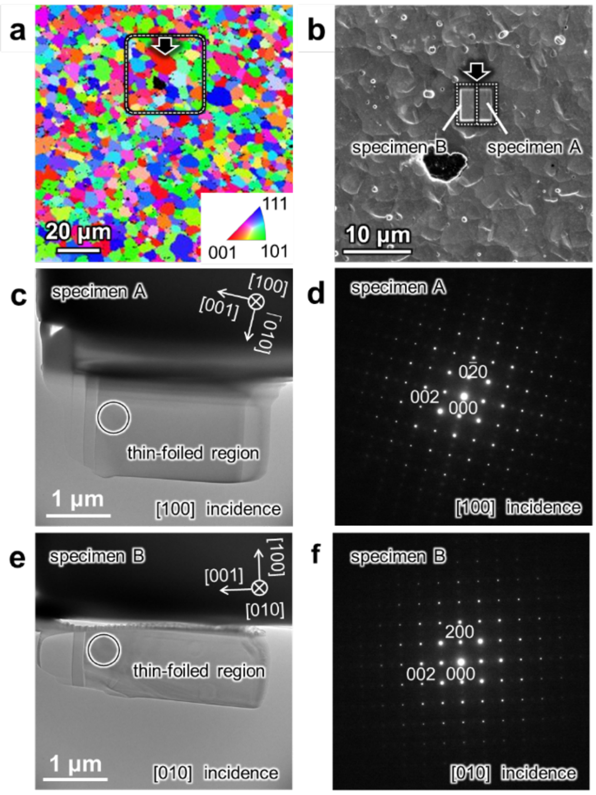

Figure S21. Identification of crystal phase by transmission electron microscopy (TEM). **a**, Orientation map of crystal grains at polished surface of dehydrated $BaSn_{0.3}Sc_{0.7}O_{3-\delta}$ electrolyte, determined by electron backscatter diffraction (EBSD). The orientations are represented by colors as per the inset of the standard triangle, assuming a perovskite-type structure. **b**, Scanning electron microscopy (SEM) image acquired from the square region in **a**. The black arrow in **b** indicates the position of the red-colored crystal grain (indicated by the arrow in **a**) in which the [001] direction was approximately vertical to the polished surface. As explained later, two specimens for TEM observations (specimens A and B) were obtained from this red-colored crystal grain, so that the crystal structure of dehydrated $BaSn_{0.3}Sc_{0.7}O_{3-\delta}$ could be analyzed with two distinct electron incidence directions of [100] and [010]. **c**, TEM image of specimen A, observed for electron incidence direction of [100]. **d**, Electron diffraction pattern obtained from circle region in **c**. The Bragg reflections were reasonably indexed with reference to the perovskite-type structure. **e**, TEM image of specimen B, observed for electron incidence direction of [010]: *i.e.,* 90° deviated from that in **c**. **f**, Electron diffraction pattern obtained from circle region in **e**. The Bragg reflections were reasonably indexed with reference to the perovskite-type structure. The observations explicitly indicate that the crystal structure is of perovskite-type with the space group $Pm\bar{3}m$, ruling out the possibilities of other structures including brownmillerite type.

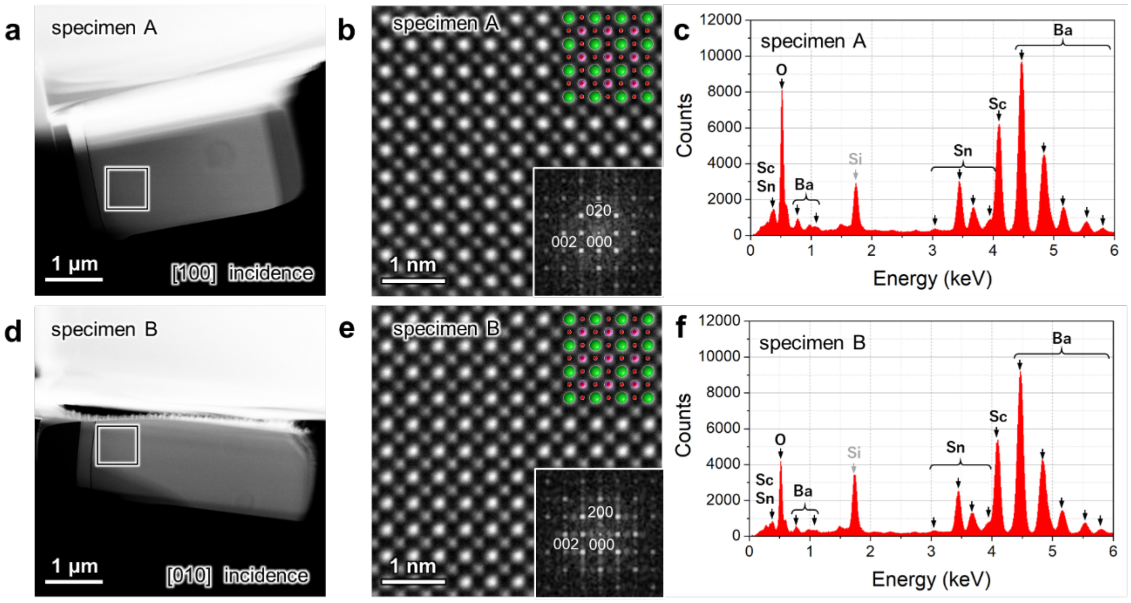

Figure S22. Direct observations of atomic arrangement and chemical composition of dehydrated $BaSn_{0.3}Sc_{0.7}O_{3-\delta}$ electrolyte. a, High-angle annular dark-field scanning transmission electron microscopy (HAADF-STEM) image of specimen A (same as that used in TEM studies in **Figure S21**). **b**, Atomic arrangement in specimen A revealed by HAADF-STEM, and digital diffractogram obtained from this image (inset). The green, pink, and red circles represent the sublattices comprising the perovskite-type structure (*i.e,* A-sites, B-sites, and O-sites) although the O-sites (occupied by oxygen) are barely observed by HAADF-STEM. **c**, Energy-dispersive X-ray spectroscopy (EDS) results for square region in **a**. **d**, HAADF-STEM image of specimen B (same as that used in TEM studies in **Figure S21**). **e**, Atomic arrangement in specimen B revealed by HAADF-STEM, and digital diffractogram obtained from this image (inset). The green, pink, and red circles represent the sublattices comprising the perovskite-type structure: refer to the caption to **b**. **f**, EDS spectrum for square region in **d**. The HAADF-STEM images indicate that the crystal structure is of perovskite type, consistent with the electron diffraction results shown in **Figure S21**. The EDS spectrum, with reference to the analyzed crystal grain, indicates that the atomic ratio of Ba : Sn : Sc is 1 : 0.30 : 0.67.

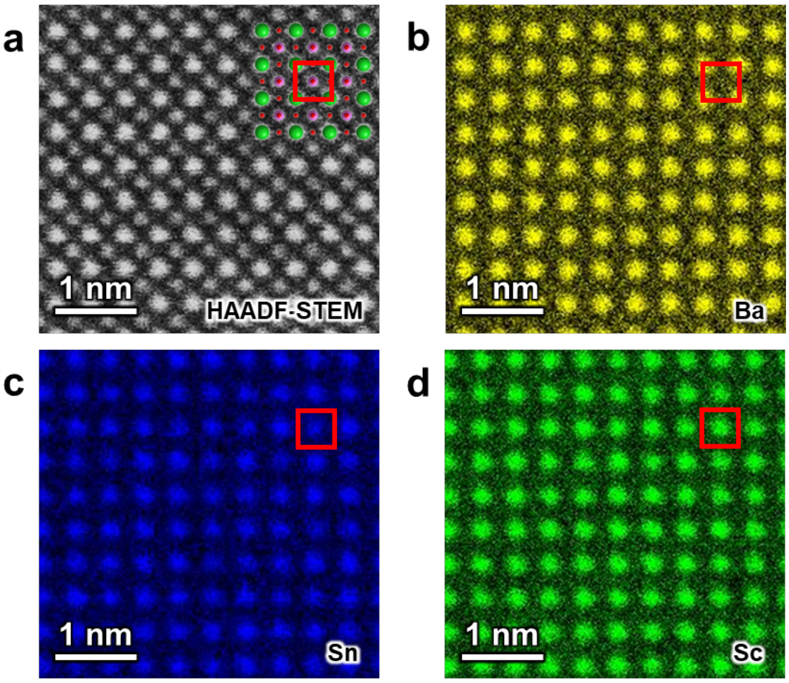

Figure S23. Sublattices comprising perovskite-type structure in dehydrated $BaSn_{0.3}Sc_{0.7}O_{3-\delta}$ electrolyte. a, HAADF-STEM image showing the atomic arrangement in dehydrated $BaSn_{0.3}Sc_{0.7}O_{3-\delta}$. The image was obtained from specimen A (refer to **Figure S22**). The green, pink, and red circles indicate the positions of sublattices in the perovskite-type structure: *i.e,* A-sites, B-sites, and O-sites, although the O-sites (occupied by oxygen atoms) are not visible in the HAADF-STEM image. The red square shows one unit cell of the perovskite-type structure. **b**, EDS map showing the presence of Ba, which occupies the A-sites. **c**, EDS map showing the presence of Sn, which occupies the B-sites. **d**, EDS map showing the presence of Sc, which occupies the B-sites. The B-sites correspond to a solid solution of Sc and Sn. The EDS maps were obtained using the Ba-K, Sc-K and Sn-K lines, respectively.

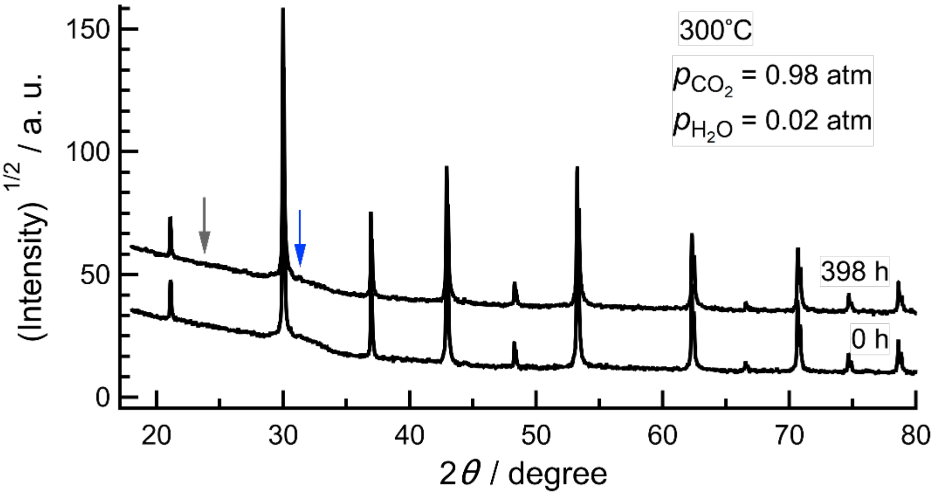

Figure S24. X-ray diffraction patterns for $BaSn_{0.3}Sc_{0.7}O_{3-\delta}$ powder before and after accelerated stability test at 300°C for 398 h under CO_2 and water partial pressures of 0.98 and 0.02 atm. The arrows at 23.7° and 31.3° indicate the expected positions of the main peaks for the $BaCO_3$ and $BaSc_2O_4$ phases, respectively; the former was not identified either before or after the test. Prior to exposure to the humidified CO_2 flow at 0 h, the powder sample was hydrated in the X-ray diffraction chamber for 9 h under a humidified N_2 flow with a water partial pressure of 0.02 atm.

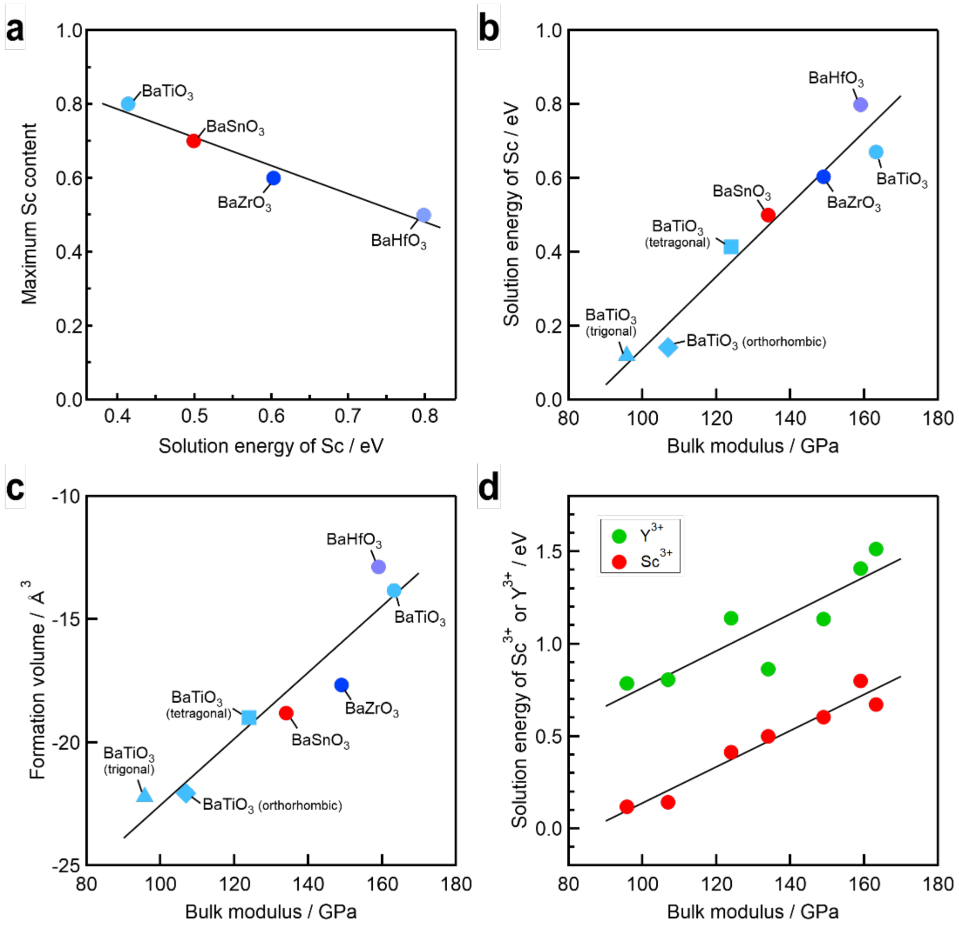

Figure S25. Solution energy for Sc and lattice softness for Ba-based perovskite oxides. **a**, Maximum Sc content against solution energy for Sc. **b**, Solution energy for Sc against bulk modulus for Ba-based perovskite oxides. **c**, Formation volume of an oxide-ion vacancy against bulk modulus. **d**, Solution energy for Sc and Y against bulk modulus for Ba-based perovskite oxides. All solution energies were calculated at the B site of perovskite-type structure. The bulk moduli were obtained from the Materials Project:[30] $BaTiO_3$, mp-5020 for R3m, mp-5777 for Amm2, mp-5986 for P4mm, and mp-2998 for Pm-3m; $BaSnO_3$, mp-3163 for Pm-3m; $BaZrO_3$, mp-3834 for Pm-3m; and $BaHfO_3$, mp-998552 for Pm-3m. The solution energy for tetragonal $BaTiO_3$ is shown in **a**, as the tetragonal phase is stable at room temperature. The low solution energies lead to higher solubility limits for Sc in $BaTiO_3$ and $BaSnO_3$ than $BaZrO_3$ in **a**.

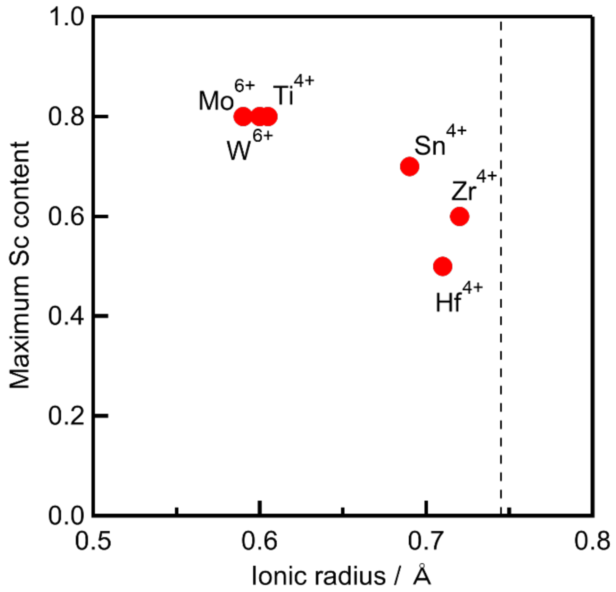

Figure S26. Maximum Sc content vs. ionic radius of B-site cation in six coordination. The dashed line indicates the ionic radius of Sc^{3+}, with ionic radii sourced from Shannon.[23] The Maximum Sc contents for Mo^{6+} and W^{6+} were assumed as 0.8 from the reports[31,32].

References

1 Yamazaki, Y. *et al.* Proton trapping in yttrium-doped barium zirconate. *Nat. Mater.* **12**, 647-651 (2013). https://doi.org/10.1038/nmat3638

2 Kresse, G. & Furthmüller, J. Efficient iterative schemes for ab initio total-energy calculations using a plane-wave basis set. *Phys. Rev. B* **54** (1996). https://doi.org/10.1103/PhysRevB.54.11169

3 Kresse, G. & Joubert, D. From ultrasoft pseudopotentials to the projector augmented-wave method. *Phys. Rev. B* **59**, 1758-1775 (1999). https://doi.org/10.1103/PhysRevB.59.1758

4 Calandrini, V., Pellegrini, E., Calligari, P., Hinsen, K. & Kneller, G. R. nMoldyn - Interfacing spectroscopic experiments, molecular dynamics simulations and models for time correlation functions. *École thématique de la Société Française de la Neutronique* **12**, 201-232 (2011). https://doi.org/10.1051/sfn/201112010

5 Michaud-Agrawal, N., Denning, E. J., Woolf, T. B. & Beckstein, O. MDAnalysis: a toolkit for the analysis of molecular dynamics simulations. *J. Comput. Chem.* **32**, 2319-2327 (2011). https://doi.org/10.1002/jcc.21787

6 Gowers, R. J. *et al.* MDAnalysis: A Python package for the rapid analysis of molecular dynamics simulations. *Proceedings of the 15th Python in Science Conference*, 98-105 (2016). https://doi.org/10.25080/Majora-629e541a-00e

7 de Buyl, P. tidynamics: A tiny package to compute the dynamics of stochastic and molecular simulations. *Journal of open source software* **3**, 877 (2018). https://doi.org/10.21105/joss.00877

8 Perdew, J. P. *et al.* Restoring the density-gradient expansion for exchange in solids and surfaces. *Phys. Rev. Lett.* **100**, 136406 (2008). https://doi.org/10.1103/PhysRevLett.100.136406

9 Ong, S. P. *et al.* Python Materials Genomics (pymatgen): A robust, open-source python library for materials analysis. *Comput. Mater. Sci.* **68**, 314-319 (2013). https://doi.org/10.1016/j.commatsci.2012.10.028

10 Kumagai, Y. & Oba, F. Electrostatics-based finite-size corrections for first-principles point defect calculations. *Phys. Rev. B* **89**, 195205 (2014). https://doi.org/10.1103/PhysRevB.89.195205

11 Freysoldt, C., Neugebauer, J. & Van de Walle, C. G. Fully ab initio finite-size corrections for charged-defect supercell calculations. *Phys. Rev. Lett.* **102**, 016402 (2009). https://doi.org/10.1103/PhysRevLett.102.016402

12 Gonze, X. & Lee, C. Dynamical matrices, Born effective charges, dielectric permittivity tensors, and interatomic force constants from density-functional

perturbation theory. *Phys. Rev. B* **55**, 10355-10368 (1997). https://doi.org/10.1103/PhysRevB.55.10355

13 Gajdoš, M., Hummer, K., Kresse, G., Furthmüller, J. & Bechstedt, F. Linear optical properties in the projector-augmented wave methodology. *Phys. Rev. B* **73**, 045112 (2006). https://doi.org/10.1103/PhysRevB.73.045112

14 Kreuer, K. D. Aspects of the formation and mobility of protonic charge carriers and the stability of perovskite-type oxides. *Solid State Ion.* **125**, 285-302 (1999). https://doi.org/10.1016/S0167-2738(99)00188-5

15 Okuyama, Y., Kozai, T., Sakai, T., Matsuka, M. & Matsumoto, H. Proton transport properties of $La_{0.9}M_{0.1}YbO_{3-\delta}$ (M=Ba, Sr, Ca, Mg). *Electrochim. Acta* **95**, 54-59 (2013). https://doi.org/10.1016/j.electacta.2013.01.156

16 Hyodo, J., Kitabayashi, K., Hoshino, K., Okuyama, Y. & Yamazaki, Y. Fast and stable proton conduction in heavily scandium-doped polycrystalline barium zirconate at intermediate temperatures. *Adv. Energy Mater.* **10**, 2000213 (2020). https://doi.org/10.1002/aenm.202000213

17 Kreuer, K. D. *et al.* Proton conducting alkaline earth zirconates and titanates for high drain electrochemical applications. *Solid State Ion.* **145**, 295-306 (2001). https://doi.org/10.1016/S0167-2738(01)00953-5

18 Yamazaki, Y., Babilo, P. & Haile, S. M. Defect chemistry of yttrium-doped barium zirconate: A thermodynamic analysis of water uptake. *Chem. Mater.* **20**, 6352-6357 (2008). https://doi.org/10.1021/cm800843s

19 Yamanaka, S. *et al.* Thermophysical properties of $BaZrO_3$ and $BaCeO_3$. *J. Alloys Compd.* **359**, 109-113 (2003). https://doi.org/10.1016/s0925-8388(03)00214-7

20 Fu, D., Itoh, M. & Koshihara, S. Y. Invariant lattice strain and polarization in $BaTiO_3$-$CaTiO_3$ ferroelectric alloys. *J. Phys. Condens. Matter.* **22**, 052204 (2010). https://doi.org/10.1088/0953-8984/22/5/052204

21 Kinyanjui, F. G. *et al.* Crystal structure and proton conductivity of $BaSn_{0.6}Sc_{0.4}O_{3-\delta}$: insights from neutron powder diffraction and solid-state NMR spectroscopy. *J. Mater. Chem. A* **4**, 5088-5101 (2016). https://doi.org/10.1039/c5ta09744d

22 Maekawa, T., Kurosaki, K. & Yamanaka, S. Thermal and mechanical properties of polycrystalline $BaSnO_3$. *J. Alloys Compd.* **416**, 214-217 (2006). https://doi.org/10.1016/j.jallcom.2005.08.032

23 Shannon, R. D. Revised effective ionic radii and systematic studies of interatomie distances in halides and chaleogenides. *Acta Crystallogr. Sect. A: Found. Crystallogr.* **32**, 751-767 (1976). https://doi.org/10.1107/S0567739476001551

24 Bjørheim, T. S., Kotomin, E. A. & Maier, J. Hydration entropy of BaZrO3 from first

principles phonon calculations. *Journal of Materials Chemistry A* **3**, 7639-7648 (2015). https://doi.org/10.1039/c4ta06880g

25 Hyodo, J., Tsujikawa, K., Shiga, M., Okuyama, Y. & Yamazaki, Y. Accelerated discovery of proton-conducting perovskite oxide by capturing physicochemical fundamentals of hydration. *ACS Energy Lett.* **6**, 2985-2992 (2021). https://doi.org/10.1021/acsenergylett.1c01239

26 Naumovska, E. *et al.* Local structure of hydrated nanocrystalline films of the proton conductor $BaZr_{1-x}Sc_xO_{3-x/2}$ studied by infrared spectroscopy. *Vib. Spectrosc* **130**, 103622 (2024). https://doi.org/10.1016/j.vibspec.2023.103622

27 Kuroha, T. *et al.* Optimum dopant of barium zirconate electrolyte for manufacturing of protonic ceramic fuel cells. *J. Power Sources* **506**, 230134 (2021). https://doi.org/10.1016/j.jpowsour.2021.230134

28 Mikami, Y., Sekitani, Y., Yamauchi, K., Kuroha, T. & Okuyama, Y. Effect of Transition Element Dissolution on Ytterbium-Doped Barium-Zirconate-Based Protonic Ceramic Fuel Cells. *ACS Applied Energy Materials* **7**, 1136-1148 (2024). https://doi.org/10.1021/acsaem.3c02674

29 Roy, T., Sahani, S., Madhu, D. & Chandra Sharma, Y. A clean approach of biodiesel production from waste cooking oil by using single phase $BaSnO_3$ as solid base catalyst: Mechanism, kinetics & E-study. *Journal of Cleaner Production* **265**, 121440 (2020). https://doi.org/10.1016/j.jclepro.2020.121440

30 Jain, A. *et al.* Commentary: The Materials Project: A materials genome approach to accelerating materials innovation. *APL Mater.* **1**, 011002 (2013). https://doi.org/10.1063/1.4812323

31 Saito, K. & Yashima, M. High proton conductivity within the 'Norby gap' by stabilizing a perovskite with disordered intrinsic oxygen vacancies. *Nat Commun* **14**, 7466 (2023). https://doi.org/10.1038/s41467-023-43122-4

32 Saito, K., Umeda, K., Fujii, K., Mori, K. & Yashima, M. High proton conduction by full hydration in highly oxygen deficient perovskite. *Journal of Materials Chemistry A* **12**, 13310-13319 (2024). https://doi.org/10.1039/d4ta01978d
